# Loss of intracellular ATP affects axoplasmic viscosity and pathological protein aggregation in mammalian neurons

**DOI:** 10.1126/sciadv.adq6077

**Published:** 2025-04-23

**Authors:** Laurent Guillaud, Anna Garanzini, Sarah Zakhia, Sandra De la Fuente, Dimitar Dimitrov, Susan Boerner, Marco Terenzio

**Affiliations:** ^1^ Molecular Neuroscience Unit, Okinawa Institute of Science and Technology Graduate University, 1919-1 Tancha, Onna-son, Kunigami-gun, Okinawa, Japan.; ^2^ Cellular and Molecular Synaptic Function Unit, Okinawa Institute of Science and Technology Graduate University, 1919-1 Tancha, Onna-son, Kunigami-gun, Okinawa, Japan.

## Abstract

Neurodegenerative diseases display synaptic deficits, mitochondrial defects, and protein aggregation. We show that intracellular adenosine triphosphate (ATP) regulates axoplasmic viscosity and protein aggregation in mammalian neurons. Decreased intracellular ATP upon mitochondrial inhibition leads to axoterminal cytosol, synaptic vesicles, and active zone component condensation, modulating the functional organization of mouse glutamatergic synapses. Proteins involved in the pathogenesis of Parkinson’s disease (PD), Alzheimer’s disease (AD), and amyotrophic lateral sclerosis (ALS) condensed and underwent ATP-dependent liquid phase separation in vitro. Human inducible pluripotent stem cell–derived neurons from patients with PD and ALS displayed reduced axoplasmic fluidity and decreased intracellular ATP. Last, nicotinamide mononucleotide treatment successfully rescued intracellular ATP levels and axoplasmic viscosity in neurons from patients with PD and ALS and reduced TAR DNA-binding protein 43 (TDP-43) aggregation in human motor neurons derived from a patient with ALS. Thus, our data suggest that the hydrotropic activity of ATP contributes to the regulation of neuronal homeostasis under both physiological and pathological conditions.

## Teaser

The protective role of ATP in neurons reduces protein aggregation and neurodegeneration.

## INTRODUCTION

Neurons transfer and store information via the propagation of electric and chemical signals between two cells through morphologically specialized junctions called synapses (*1*). Thus, the morphological and functional organization of the presynaptic compartment is paramount to maintain both cognitive and motor functions (*2*). Liquid phase separation (LPS) has recently emerged as an essential mechanism to regulate neuronal development (*3*) and the subcellular organization and function of synapses (*4*). Various proteins of the presynaptic release machinery, such as synapsin-1 (SYN1) (*5*), the RAB3a-interacting molecule (RIM), and RIM-binding proteins (*6*) in mammalian neurons, as well as the scaffolding protein liprin-α homologue (SYD-2) and the glutamine, leucine, lysine, and serine-rich protein-1 (ELKS-1) (*7*) in *Caenorhabditis elegans* neurons, have been shown to undergo LPS in vitro. The essential role of LPS in the organization and function of the presynaptic compartment have been recently highlighted by a computational analysis of around 500 presynaptic proteins, with more than 80 showing a high probability of phase separation (*8*).

Synaptic dysfunctions are pathological hallmarks in both early and late stages of neurodegenerative diseases. Altered neuronal excitability, excitotoxicity, or aberrant synapse formation have been reported in both cognitive disorders such as Parkinson’s disease (PD) and Alzheimer’s disease (AD) (*9*) and motor disorders such as amyotrophic lateral sclerosis (ALS) (*10*). Dysregulation of synaptic gene expression, perturbations of synaptic vesicle (SV) cycle and synaptic transmission, and a reduction in the number of synapses have been reported in α-synuclein (SNCA) human inducible pluripotent stem cell (hiPSC) models of PD (*11*), in amyloid-β precursor protein (APP) hiPSC models of AD (*12*–*15*), and in a zebrafish genetic model of ALS (*16*).

Formation of membrane-less protein aggregates by LPS is also a prominent feature of neurodegenerative disorders (*17*–*19*), and pathological aggregation and toxicity of Tau in AD, fused in sarcoma (FUS) or TAR DNA-binding protein 43 (TDP-43) in ALS, and huntingtin in Huntington’s disease have been directly linked to their LPS (*20*–*24*). Similar mechanisms have also been proposed for the aggregation and toxicity of APP and SNCA in PD (*25*, *26*). Although LPS has been extensively studied in vitro, identifying the molecular triggers underlying LPS initiation, and the formation of highly condensed protein assembly remains challenging. Recently, it was shown that adenosine triphosphate (ATP) can act as a potent hydrotropic agent, regulating protein aggregation in vitro (*27*, *28*). Thus, in addition to its energetic function, ATP might play an essential role in the regulation of protein solubility in vivo, as suggested in *Xenopus* oocytes, where the injection of ATP induced a complete solubilization of nucleophosmin (Npm1) aggregates present in nucleoli (*29*).

Under homeostatic conditions, mitochondria are the main organelles producing ATP (*30*), and mitochondrial dysfunctions are often associated with neurodegenerative diseases (*31*) and aging (*32*). Numerous morphological and functional alterations of mitochondria have been reported in the pathogenesis of SNCA and parkin (PARK2 or PARK7) mutant hiPSC models of PD (*33*–*36*), in models of AD (*37*, *38*), and in TDP-43, FUS, and superoxide dismutase 1 (SOD1) mutant models of ALS (*39*). Mitochondrial function is highly dependent on the oxidized form of nicotinamide adenine dinucleotide (NAD^+^), which plays a key role in cellular energy metabolism and energy production (*40*). NAD^+^ precursors such as nicotinamide mononucleotide (NMN) and nicotinamide riboside (NR) have been reported to improve energy activity and neuronal survival in various animal models of neurodegenerative diseases (*41*–*44*).

Here, we found a robust correlation between mitochondrial activity, local ATP concentration, and viscoadaptation of the presynaptic release machinery in cultured rodent neurons. Next, we used an in vitro assay to show how key proteins involved in PD, AD, and ALS undergo LPS and form condensates in the absence of ATP, while the presence of ATP prevents their condensation and promotes the decondensation of preformed aggregates. We also looked at the solubility of axonal cytosol in hiPSC-derived neurons from patients with PD and ALS, both displayed significant reductions in intracellular levels of ATP and in axoplasmic viscosity compared to healthy controls. Last, activation of NAD^+^ by NMN treatment successfully rescued intracellular levels of ATP and axoplasmic fluidity and reduced the pathological aggregation of TDP-43 in motor neurons derived from a patient with ALS and the TDP-43^N390D^ mutation. Thus, our data emphasize the role of ATP as a key regulator of LPS in mammalian neurons and suggest that the production of ATP by active mitochondria might be a critical and conserved mechanism for the regulation of axosynaptic organization and function, as well as in mitigating protein aggregation associated with neurodegenerative diseases.

## RESULTS

We used the mouse giant calyceal glutamatergic synapse culture model (*45*), previously shown to be well suited for live-imaging experiments (*46*), to analyze presynaptic viscosity by real-time confocal microscopy. Since a large number of presynaptic proteins might undergo LPS (*8*) and potentially affect cytosol fluidity, we chose to estimate the level of protein aggregation in the axoterminal compartment by performing a global assessment of the axoplasmic fluidity using fluorescence recovery after photobleaching (FRAP) of cytosolic green fluorescent protein (cGFP) rather than probing candidate proteins individually. cGFP has been shown to remain highly soluble in cell cytoplasm, with less than 2% localizing to organelles (*47*). In addition, FRAP has been a method of choice to study LPS both in vitro and in vivo (*18*, *48*) and used to measure cytosol viscoadaptation in live yeast cells upon energy deprivation (*49*, *50*). The percentage of fluorescence recovery, representative of the diffusion of cGFP, provides an estimation of the fluidic properties of the cytosol, with higher recovery suggesting higher solubility (solute phase) and lower recovery indicating lower solubility (condensate phase). Thus, we used the diffusion of cGFP as a proxy to estimate the global modifications in axonal cytosolic viscosity associated with protein aggregation (fig. S1).

### Heterogeneous mitochondrial activity modulates presynaptic cytosol fluidic phase

Presynaptic terminals are highly enriched in mitochondria, as synaptic transmission relies extensively on ATP (*51*). We visualized active mitochondria using tetramethyl rhodamine ester (TMRE) and assessed the fluidity of the presynaptic cytosol in different regions of mouse calyceal terminals (mCTs) by bleaching the cGFP signal locally and monitoring its recovery over time. Our FRAP analysis in mCTs (fig. S2A) revealed distinct fluorescence recovery profiles in different regions of mCTs, where the regions below 60% fluorescence recovery appeared not to contain any active mitochondria, while active mitochondria localized in regions above 80% fluorescence recovery (fig. S2, C and D). These data suggest that the presence of active mitochondria might contribute to the mechanisms underlying presynaptic cytosolic viscoadaptation.

### Blocking mitochondrial activity reduces the solubility of the presynaptic release machinery

**Fig. 1.**
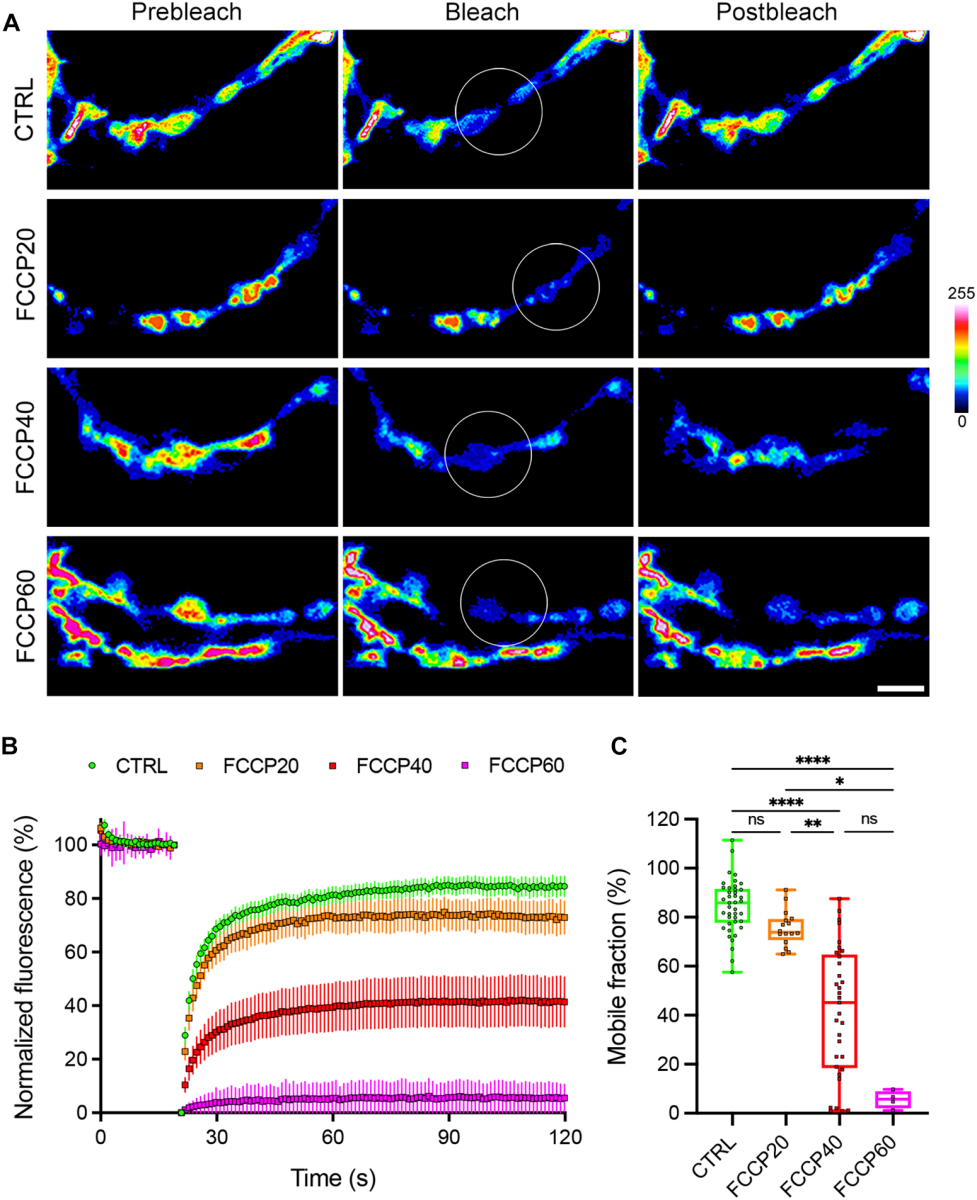
Inhibition of mitochondrial activity reduces presynaptic cytosol fluid phase. (**A**) Untreated (CTRL) mCTs and mCTs treated with FCCP for 20 min (FCCP20), 40 min (FCCP40), and 60 min (FCCP60), expressing cGFP (pseudocolor). FRAP was performed on a region of interest (ROI) containing at least one calyceal swelling (white circle). Sequential images show the ROI before bleach (left), during bleach (middle), and after recovery (right). Scale bar, 2 μm; color bar, cGFP fluorescence intensity. (**B**) Fluorescence intensity recovery profiles in control (*n* = 41 cells from eight independent experiments) mCTs and in mCTs treated with FCCP for 20 min (*n* = 17 cells from four independent experiments), 40 min (*n* = 33 cells from seven independent experiments), and 60 min (*n* = 4 cells from four independent experiments). Means ± 95% confidence interval (CI). (**C**) Mobile cGFP fraction representing cytosolic fluidity estimated from the past 20 s of the fluorescence intensity profile in control mCTs and mCTs treated with FCCP for 20 and 40 min. Box plot with median and minimum/maximum whiskers. Kruskal-Wallis nonparametric test with Dunn’s correction. ns, not significant. *P = 0.0209, **P = 0.0081, and ****P < 0.0001.

To further assess whether local ATP production affects the presynaptic cytosolic viscosity, we blocked mitochondrial activity by incubating calyceal terminals expressing cGFP with 50 μM carbonyl cyanide *p*-trifluoromethoxyphenylhydrazone (FCCP) for 20, 40, and 60 min at 37°C. FRAP analysis of untreated or FCCP-treated mCTs showed ~1.2-, ~2-, and ~15-fold reductions in cGFP fluorescence recovery after FCCP treatment, respectively, compared to untreated mCTs (Fig. 1, A and B). We limited our observations to 40 min after FCCP addition, as extended exposure (1 hour or more) led to almost complete inhibition of fluorescence recovery, probably resulting from cell toxicity. However, we did not observe any significant increase in cell death after 40 min of FCCP treatment compared to untreated samples (fig. S3, A and B), suggesting that our observations are likely the result of energy depletion rather than cell toxicity. The mobile fraction of cGFP was estimated from the past 20 s of the fluorescent profile in both control and FCCP-treated mCTs and showed a strong reduction upon FCCP treatment compared to control, suggesting a 50% decrease in cGFP fluidity 40 min after blocking mitochondrial activity (Fig. 1C). We also incubated mCTs with 20 μM rotenone, another mitochondrial activity blocker, and observed a ~1.2-fold reduction in cGFP fluorescence recovery and diffusion (fig. S4, A and B). Conversely, blocking glycolysis by incubating mCTs with 2 mM 2-deoxyglucose (2-DG) did not significantly affect cGFP fluorescence recovery compared to control terminals (fig. S4, A and B). In addition, similar observations were recorded in control mCTs overexpressing cytosolic FusionRed instead of GFP (fig. S4, A and B), indicating that the cytosolic viscosity was not affected by the fluorescent probe used. These observations suggest that mitochondria are mainly responsible for ATP production at presynaptic sites and that their activity is paramount to maintain presynaptic cytosol fluidity. In addition, presynaptic axons incubated with 20 μM oligomycin (ATP synthase inhibitor) showed a ~1.3-fold decrease in cGFP recovery comparable to the reduction observed upon FCCP incubation (fig. S4, C and D).

Next, we confirmed whether blocking mitochondrial activity with FCCP affects the diffusion of SVs and active zones (AZs), two components of the presynaptic release machinery known to undergo LPS (*5*). We expressed Venus-vesicular glutamate transporter 2 (Venus-VGLUT2) (fig. S5A) or mTurquoise2-RIM1 (fig. S5D) in mCTs to label and assess SV pools and AZ fluidity, respectively. FCCP-treated mCTs showed a ~1.9- and ~2.5-fold reduction in Venus-VGLUT2 and mTurquoise2-RIM1 fluorescence recovery, respectively (fig. S5, B, C, E, and F). Reduction in mitochondrial activity induced by FCCP did not significantly affect the movement of SVs between calyceal swellings compared with untreated terminals, but significantly impaired the movement of SVs within calyceal swellings (fig. S6). The first process has been shown to rely on ATP-dependent active transport, while the second process mainly relies on passive diffusion (*46*). Thus, the decrease in ATP production induced by blocking mitochondrial activity appears to be sufficient to affect the viscosity of the presynaptic compartment without significantly altering other energy-dependent mechanisms. Together, these data indicate that the fluidity of the presynaptic cytosol, SV pools, and AZ components is tightly modulated by mitochondria-dependent production of ATP in the terminals.

### Mitochondrial activity and ATP concentration regulate the condensation state of cytosolic aggregates

**Fig. 2.**
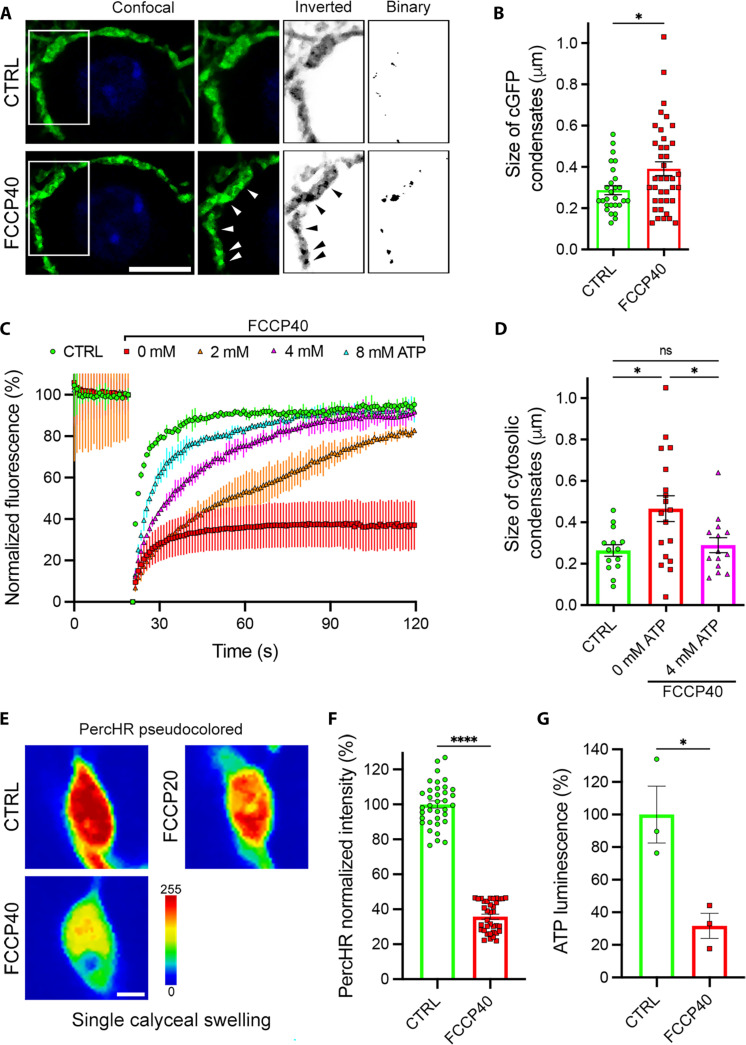
Mitochondrial activity and adenosine triphosphate (ATP) regulate the condensation of cytosolic aggregates in calyceal terminals. (**A**) Untreated (CTRL) calyceal terminals (mCTs) and mCTs treated with FCCP for 40 min expressing cGFP (green) and labeled with NucBlue (blue). Arrowheads highlight cGFP cytosolic aggregates. Scale bar, 10 μm. (**B**) Size of cytosolic condensates in control (*n* = 27 from three independent experiments) and FCCP-treated (*n* = 40 from three independent experiments) mCTs. Means ± SEM, unpaired Student’s *t* test. *P = 0.0215. (**C**) Fluorescence intensity recovery profiles in control (*n* = 14 from three independent experiments), FCCP-treated (*n* = 18 cells from four independent experiments), FCCP-treated + 2 mM ATP (*n* = 12 cells from three independent experiments), FCCP-treated + 4 mM ATP (*n* = 14 from four independent experiments), and FCCP-treated + 8 mM ATP (*n* = 9 from three independent experiments) mCTs. Means ± 95% CI. (**D**) Size of cytosolic condensates in control (*n* = 14 from three independent experiments), FCCP-treated (red, *n* = 18 from four independent experiments), and FCCP-treated +4 mM ATP (*n* = 14 from four independent experiments) mCTs. Means ± SEM, one-way analysis of variance (ANOVA) with Tukey’s correction. *P = 0.0137 and 0.0349. (**E**) Untreated (CTRL) mCTs and mCTs treated with 20- and 40-min FCCP, expressing PercevalHR (PercHR). Scale bar, 1 μm; color bar, PercevalHR intensity. (**F**) Quantification of PercevalHR fluorescence intensity in control (*n* = 36 from three independent experiments) and FCCP-treated (*n* = 36 from three independent experiments) mCTs. Means ± SEM, unpaired Student’s *t* test. ****P < 0.0001. (**G**) In vitro bioluminescence measurement of intracellular ATP in control and FCCP-treated mCTs from three independent samples. Means ± SEM, unpaired Student’s *t* test. *P = 0.0230.

To further characterize presynaptic cytosol fluidity, we visualized and quantified the formation of cytosolic aggregates concentrating cGFP in control and FCCP-treated mCTs. FCCP treatment promoted the spatial restriction of cGFP-containing cytosolic aggregates in calyceal terminals and their increase in size compared to untreated terminals (Fig. 2, A and B). We were able to rescue cGFP fluidity by chitosan-assisted delivery of increasing amounts of ATP into mCTs before FCCP treatment. ATP (4 to 8 mM) completely rescued cGFP fluorescence recovery to the levels observed in untreated terminals (Fig. 2C). In addition, a concentration of 4 mM ATP significantly reduced the formation and size of cGFP-containing cytosolic aggregates in calyceal terminals treated with FCCP (Fig. 2D). We did not observe significant change in intracellular pH upon chitosan-assisted delivery of 4 mM ATP in ventral cochlear nucleus (VCN) neurons (fig. S7, A and B), suggesting a direct effect of the hydrotropic activity of ATP rather than resulting from a pH shift.

Next, we tried to evaluate the local decrease in ATP associated with the FCCP-induced reduction of mitochondrial activity by overexpressing the ATP/adenosine diphosphate (ADP) fluorescent sensor PercevalHR in mCTs (fig. S8, A and B). We observed that PercevalHR fluorescence varied in accordance with changes in TMRE fluorescence intensity (fig. S8, C and D). Our data showed a strong correlation between PercevalHR and TMRE fluorescence (fig. S8E), indicating that PercevalHR reliably reported local changes in ATP concentration. Upon FCCP treatment, we observed a ~65% decrease in PercevalHR fluorescence (Fig. 2, E and F), suggesting a ~2.8-fold reduction in ATP concentration after blocking mitochondrial activity. We confirmed a ~3.1-fold decrease in ATP in FCCP-treated compared to untreated mCTs by measuring ATP levels using an in vitro luminescence assay (Fig. 2G).

Thus, blocking mitochondrial activity leads to a two- to threefold decrease in intracellular ATP concentration and triggers cytosolic protein aggregation and reduction in cytosolic viscosity. Conversely, an ATP concentration of 4 mM or higher can rescue cytosolic protein solubility in presynaptic terminals.

### ATP regulates the aggregation/disaggregation of proteins involved in neurodegenerative diseases in vitro

**Fig. 3.**
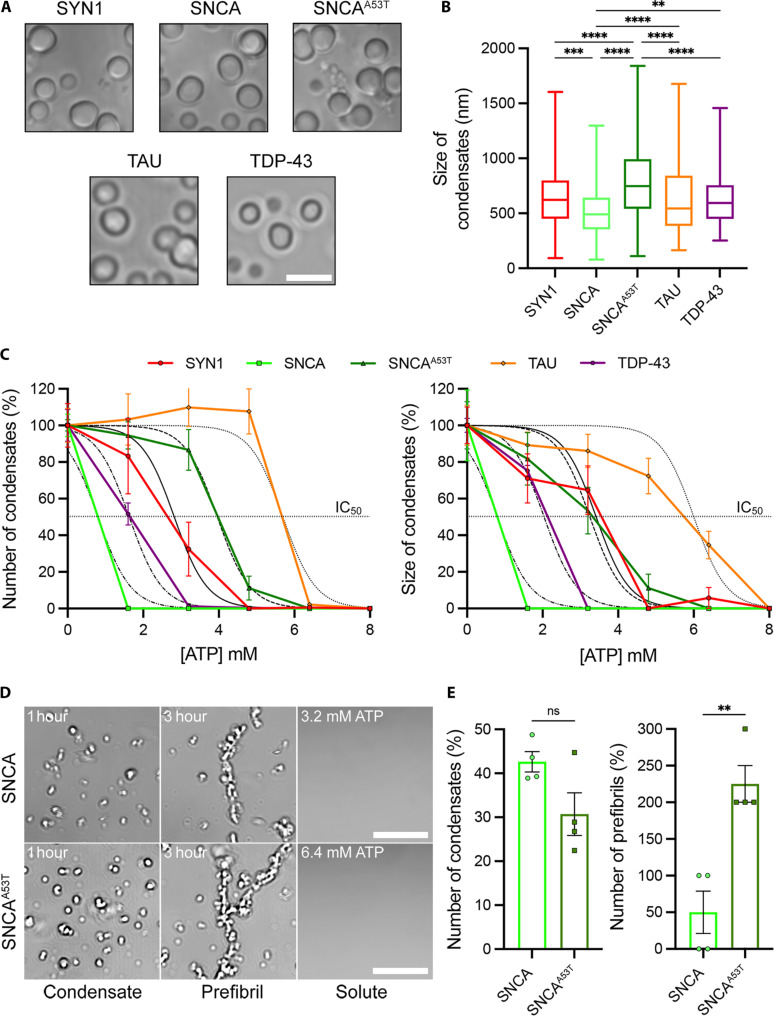
Adenosine triphosphate (ATP) regulates neuronal protein liquid phase separation (LPS) in vitro. (**A**) Liquid phase–separated condensates formed after 1 hour at 37°C from 10 μM protein in Tyrode’s solution and without crowding agents. Scale bar, 1 μm. (**B**) Estimated size of protein condensates obtained from binary images [*n* = 278, 151, 247, 280, and 268 aggregates for SYN1, SNCA, SNCA^A53T^ mutant, Tau (TAU), and TDP-43, respectively]. Box plot with median and minimum/maximum whiskers. Kruskal-Wallis nonparametric test with Dunn’s correction for multiple comparison. **P = 0.0064, ***P = 0.0002, and ****P < 0.0001. (**C**) Proportion of remaining condensates (left) and size of remaining condensates (right) after adding increasing concentrations of ATP ranging from 2 to 8 mM. Means ± SEM (pooled from four independent experiments). IC_50_, half-maximal inhibition concentration. (**D**) Representative images of SNCA (top) and SNCA^A53T^ mutant (bottom) protein condensates and prefibrils after 1 to 3 hours of incubation at 37°C and after decondensation by 3.2 and 6.4 mM ATP, respectively. Scale bar, 5 μm. (**E**) Proportion of SNCA and SNCA^A53T^ condensates (left; *n* = 202 and 244 condensates, respectively) and prefibrils (right; *n* = 4 and 11 prefibrils, respectively) after 3 hours of incubation at 37°C. Data collected and pooled from four independent experiments. Unpaired Student’s *t* test. **P = 0.0038.

Next, we performed an in vitro LPS assay using purified recombinant proteins (fig. S9A) known to be involved in neuronal function and neurodegeneration, such as SYN1, SNCA and SNCA^A53T^ mutant, Tau, TDP-43, APP, Parkin (PARK2), and LIN28 (*5*, *17*, *52*). We observed a shift in the migration of SYN1 to a higher molecular weight than expected; thus, we confirmed the identity of the protein by Western blot using a polyclonal antibody against SYN1 (fig. S9B). We asked clarifications to the vendor, including the use of a cMyc/DKK tag on the SYN1 protein. A problem in the denaturation of the protein or a difference under the gel electrophoresis conditions were possible explanations given for this unexpected shift. At a final concentration of 10 μM, all these proteins in solution were able to phase-separate and form condensates after 1 hour at 37°C in the absence of a crowding agent (Fig. 3A and fig. S10A), as previously reported for SYN1 (*5*). The average condensate diameter for each protein was estimated, with smaller condensates being observed for PARK2 and SNCA, intermediate for APP and LIN28, and larger for TDP-43, SYN1, Tau, and SNCA^A53T^ (Fig. 3B and fig. S10, A, B, and D). We then tested whether adding increasing amounts of ATP could promote the solubilization of preexisting condensates. We observed that SNCA^A53T^, Tau, and PARK2 condensates required higher ATP concentration (4 to 8 mM) to solubilize, while SYN1, SNCA, APP, TDP-43, and LIN28 condensates solubilized between 1.6 and 3.2 mM ATP (Fig. 3C and figs. S10C and S11). Unexpectedly, the size of remaining PARK2 and LIN28 condensates did not decrease significantly after the addition of ATP (figs. S10C and S11). We next tested whether the presence of ATP could prevent the formation of protein aggregates and found that ATP inhibits the initial formation of condensates (fig. S12). We then focused on SNCA as the A53T mutation is a well-known marker of PD (*53*). SNCA^A53T^ formed the largest aggregates (Fig. 3, A and B) and required higher ATP concentration to decondense (Fig. 3C and fig. S11). Increasing the incubation time of SNCA and SNCA^A53T^ from 1 to 3 hours resulted in the formation of elongated structures (Fig. 3D) resembling SNCA protofibrils found in PD (*54*), which could be formed by the fusion of SNCA and SNCA^A53T^ condensates or a mixture of both. However, SNCA^A53T^ protofibrils were more abundant (4.5-fold) and required twice the concentration of ATP to solubilize compared to wild-type SNCA protofibrils (Fig. 3E). Notably, we did not observe any significant fibrillar formations for other proteins. We also confirmed the condensate nature of these phase-separated droplets by measuring the fluorescence recovery of Alexa Fluor 488–labeled SNCA^A53T^ after photobleaching in vitro (fig. S13, A to C). Fluorescently labeled SNCA^A53T^ condensates also retained their sensitivity to ATP, as demonstrated by the disappearance of preexisting fluorescent condensates upon the addition of 8 mM ATP (fig. S13D) and the absence of fluorescent condensate formation in the presence of 8 mM ATP during the initial 1-hour incubation period (fig. S13E). Since SYN1 condensates have been shown to recruit SNCA (*55*) and maintain SV clusters in synaptic terminals (*56*, *57*), we tested whether the addition of SNCA or SNCA^A53T^ could affect the sensitivity of SYN1 condensates to ATP in vitro. The sensitivity of SYN1/SNCA condensates to ATP was comparable to SYN1 alone, while SYN1/SNCA^A53T^ condensates leaned toward an increase requirement for ATP to solubilize (fig. S13F).

**Fig. 4.**
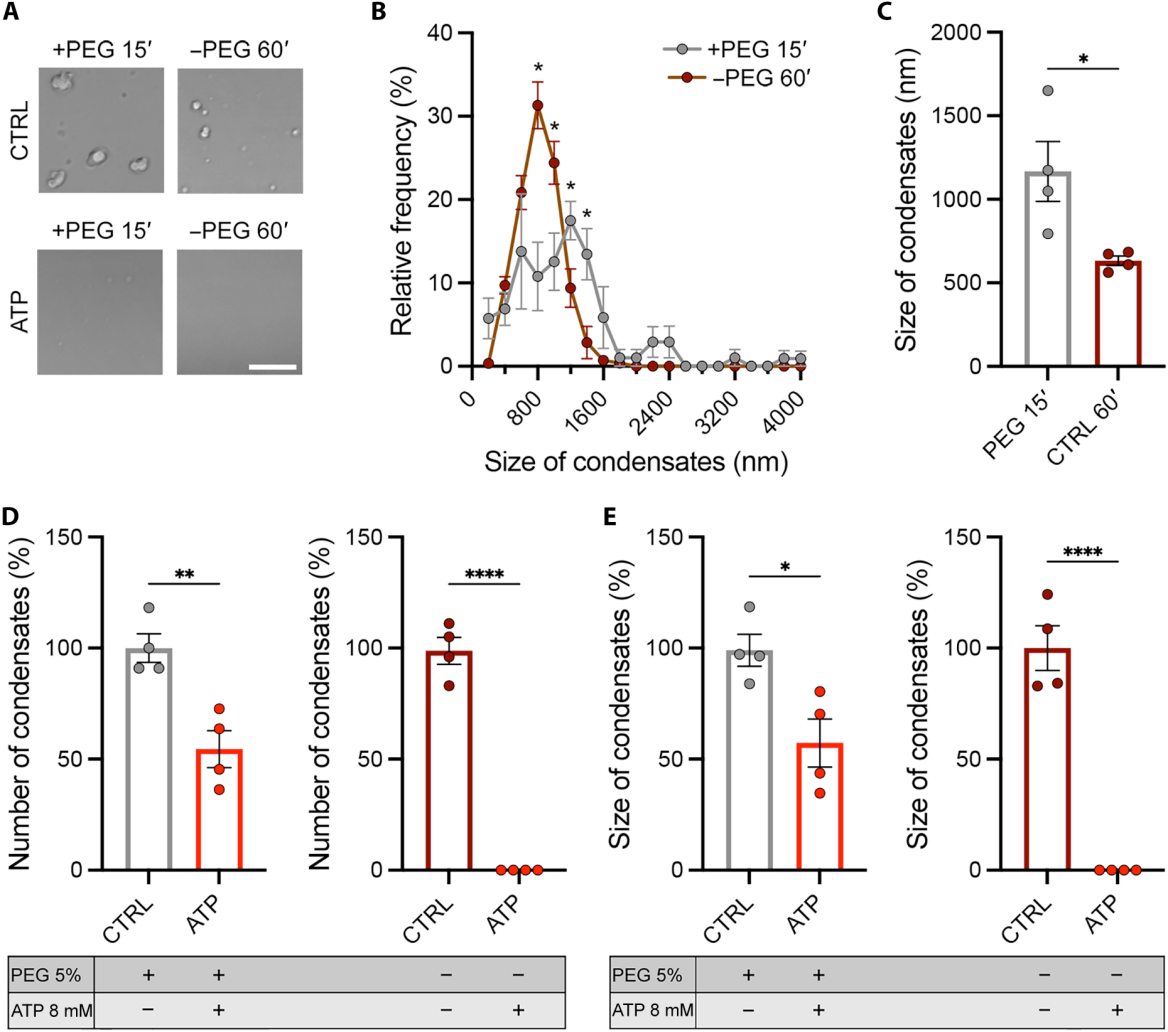
adenosine triphosphate (ATP)-dependent decondensation of SYN1 droplets preformed in the presence of polyethylene glycol (PEG). (**A**) Representative images of liquid phase–separated SYN1 condensates formed in presence of 5% PEG and after 60′ without PEG at 37°C from 10 μM protein solutions under control condition (top) or after the addition of 8 mM ATP (bottom). Scale bar, 5 μm. (**B**) Distribution of SYN1 droplet sizes with and without PEG. Means ± SEM (pooled from four independent experiments), multiple unpaired Student’s *t* test. *P = 0.0061, 0.0326, 0.0482, and 0.0265. (**C**) Average size of SYN1 droplets with and without PEG. Means ± SEM (pooled from four independent experiments), unpaired Student’s *t* test. *P = 0.0258. (**D**) Normalized number of SYN1 droplets with and without PEG under control conditions or after the addition of ATP. Means ± SEM (pooled from four independent experiments), unpaired Student’s *t* test. **P = 0.0049 and ****P < 0.0001. (**E**) Normalized size of SYN1 droplets with and without PEG under control conditions or after the addition of ATP. Means ± SEM (pooled from four independent experiments), unpaired Student’s *t* test. *P = 0.0182 and ****P < 0.0001.

Last, we assessed whether the addition of a crowding agent would affect the formation of protein condensates and their sensitivity to ATP. As previously reported, SYN1 can phase-separate in absence of polyethylene glycol (PEG) after 1 hour of incubation at a concentration ranging from 0.5 to 20 μM, while the addition of a crowding agent, commonly used to generate phase diagrams, promotes faster formation of larger condensates (*5*). Similarly, we also showed that the addition of 5% PEG to the various protein solutions at 10 μM induced rapid formation of larger droplets within 15 min, compared to the 60 min required in the absence of PEG for SYN1 (Fig. 4, A to C), SNCA^A53T^ (fig. S14, A to C), and TDP-43 (fig. S15, A to C). As expected and similarly to what we found in the absence of PEG, the addition of 8 mM ATP to the protein/PEG solution reduced the number and size of SYN1 (Fig. 4, A, D, and E), SNCA^A53T^ (fig. S14, A, D, and E), and TDP-43 (fig. S15, A, D, and E) condensates, although to a lesser extent than what we observed in protein solution without PEG. Thus, while protein condensates formed in the presence of PEG seem more resistant, they are still susceptible to ATP-dependent decondensation.

Together, these results suggest that ATP effectively regulates the formation of phase-separated aggregates in vitro and that this mechanism resembles the aggregation of SNCA, Tau, or TDP-43 as described in PD, AD, and ALS, respectively. In addition, while the LPS of most of the proteins presented here has already been reported, this work provides evidence of PARK2 LPS in vitro and confirms the recent report on LIN28 LPS (*58*).

### Cytosolic fluidity is reduced in axons from PD and ALS hiPSC-derived neurons

**Fig. 5.**
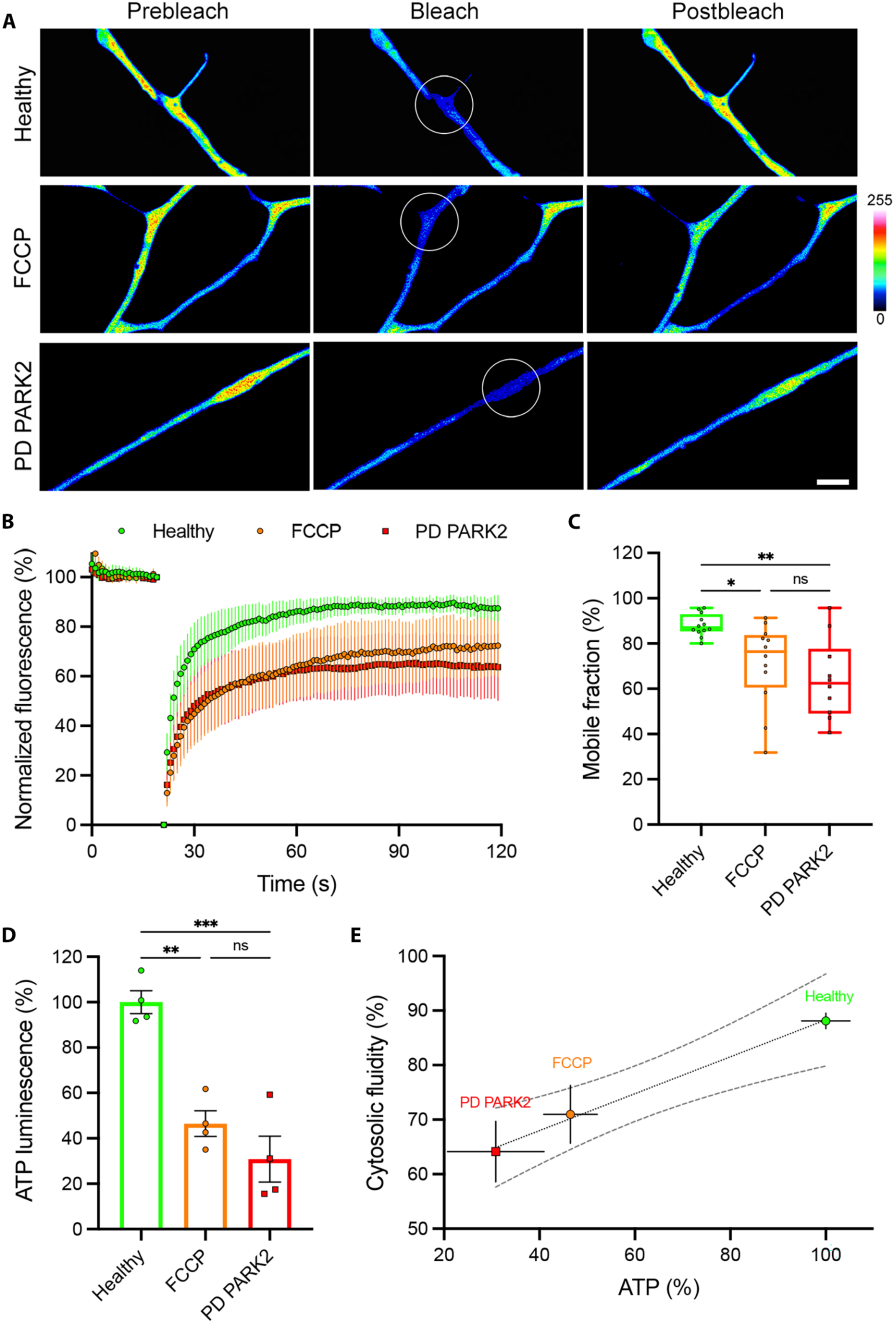
Axonal cytosol fluidity and intracellular adenosine triphosphate (ATP) are reduced in PD PARK2 hiPSC-derived neurons. (**A**) hGN axons from a healthy individual, a healthy individual treated with 50 μM FCCP for 40 min, or a patient with PD PARK2 patient, expressing cGFP (pseudocolor). FRAP ROI before bleaching, during bleach, and after recovery is shown. Scale bar, 2 μm; color bar, cGFP fluorescence intensity. (**B**) Fluorescence intensity recovery profiles of healthy hGNs, FCCP-treated healthy hGNs, and PD PARK2 hGNs. Means ± 95% CI. (**C**) Mobile cGFP fraction representing cytosolic fluidity estimated from the past 20 s of the fluorescence intensity profile of healthy control (*n* = 12 cells from three independent experiments), FCCP-treated (*n* = 12 cells from three independent experiments), and PD (*n* = 10 cells from three independent experiments) hGNs. Box plot with median and minimum/maximum whiskers. Kruskal-Wallis nonparametric test with Dunn’s correction. *P = 0.0269 and **P = 0.0038. (**D**) In vitro bioluminescence measurement of intracellular ATP in healthy hGNs, FCCP-treated healthy hGNs, and PD PARK2 hGNs (*n* = 4 samples from four independent experiments). Means ± SEM, one-way ANOVA with Tukey’s correction. **P = 0.0015 and ***P = 0.0002. (**E**) Correlation between intracellular level of ATP and cytosolic fluidity from healthy hGNs, FCCP-treated healthy hGNs, and PD PARK2 hGNs. Means ± SEM with simple linear regression (dotted line; *r*^2^ = 0.32) and 95% CI (dashed lines).

**Fig. 6.**
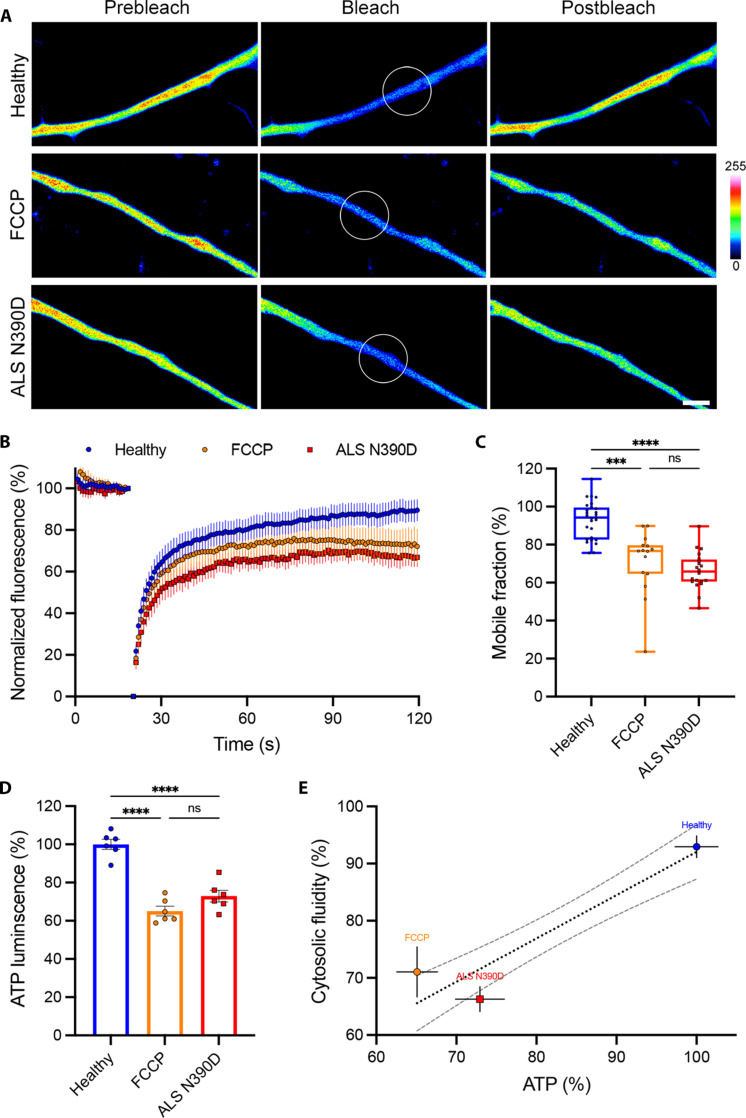
Axonal cytosol fluid phase and intracellular adenosine triphosphate (ATP) are reduced in amyotrophic lateral sclerosis (ALS) TDP-43^N390D^ hiPSC-derived neurons. (**A**) ALS hMN axons from a healthy individual, a healthy individual treated with FCCP for 40 min, or a patient with ALS TDP-43^N390D^ expressing cGFP (pseudocolor). FRAP ROI before bleach, during bleach, and after recovery. Scale bar, 2 μm; color bar, cGFP fluorescence intensity. (**B**) Fluorescence intensity recovery profiles of healthy hMNs, FCCP-treated healthy hMNs, and ALS N390D hMNs. Means ± 95% CI. (**C**) Mobile cGFP fraction representing cytosolic fluidity estimated from the past 20 s of the fluorescence intensity profile of healthy control (*n* = 27 cells from three independent experiments), FCCP-treated healthy hMNs (orange, *n* = 15 cells from three independent experiments), and ALS hMNs (*n* = 20 cells from three independent experiments). Box plot with median and minimum/maximum whiskers. Kruskal-Wallis nonparametric test with Dunn’s correction. ***P = 0.0002 and ****P < 0.0001. (**D**) In vitro bioluminescence measurement of intracellular ATP in healthy hMNs, FCCP-treated healthy hMNs, and ALS hMNs (*n* = 6 samples collected from three independent experiments). Means ± SEM, one-way ANOVA with Tukey’s correction. ****P < 0.0001. (**E**) Correlation between intracellular level of ATP and cytosolic fluidity of healthy control hMNs, FCCP-treated healthy hMNs, and ALS N390D hMNs. Means ± SEM with simple linear regression (dotted line; *r*^2^ = 0.47) and 95% CI (dashed lines).

We showed how cytosolic protein aggregation is regulated by ATP production from active mitochondria in mouse central synapses. We also described how the propensity of several proteins found in human neurodegenerative diseases to condense can be modulated by ATP concentration in vitro. To test whether ATP-dependent LPS might be a conserved mechanism leading to protein aggregation in human neurodegenerative diseases, we measured the axonal cytosolic fluidity of neurons from patients with PD and ALS. Human glutamatergic neurons (hGNs) and human motor neurons (hMNs) generated from pluripotent stem cells derived from healthy individuals or patients with PD and ALS were transfected with cGFP (fig. S16, A and B), and their axoplasmic viscosity was analyzed by FRAP. Axons from patients with PARK2 PD showed a ~1.5-fold reduction in cGFP fluorescence recovery (Fig. 5, A and B) and a significant reduction in their mobile fraction compared to healthy controls (Fig. 5C). Similarly, axons from patients with TDP-43^N390D^ ALS showed a ~1.4-fold reduction in cGFP fluorescence recovery (Fig. 6, A and B) and a significant reduction in their mobile fraction compared to healthy controls (Fig. 6C). The aforementioned observations suggest an alteration of axonal ATP concentration in patients’ neurons. To test this hypothesis, we used an in vitro luminescence assay to measure ATP concentration in our neuronal cultures and observed a ~3.2-fold reduction in the intracellular levels of ATP in PARK2 PD hGNs compared to healthy controls (Fig. 5D), as well as a ~1.4-fold decrease in ATP in TDP-43^N390D^ ALS hMNs compared to healthy controls (Fig. 6D). We uncovered substantial correlation between the intracellular levels of ATP and the axoplasmic viscoadaptation in both hGNs (Fig. 5E) and hMNs (Fig. 6E). The decrease in cytosolic fluidity and intracellular levels of ATP observed in both PD and ALS neurons were in range with the decrease observed in healthy neurons treated with FCCP (Figs. 5, C and D, and 6, C and D).

To further validate our cGFP FRAP observations, we tracked the displacement of fluorescent nanobeads in purified cytosolic fractions from control or energy-depleted neurons, where mitochondrial activity was impaired by incubation with 50 μM FCCP for 40 min. We compared the diffusion coefficient of the nanobeads under different conditions, which showed a significant decrease in energy-deprived neuronal cytoplasm (fig. S17, A to C). The decrease in cytosolic fluidity upon energy deprivation measured by FRAP readouts in live neurons was in good agreement with our microrheology measurements obtained on purified cytosolic fraction (fig. S17D), confirming a direct hydrotropic effect of ATP on cytosol viscoadaptation rather than unspecific cGFP and membrane interaction. Furthermore, a similar decrease in cytosolic fluidity and intracellular levels of ATP was also recorded in one additional PD line from a patient with SNCA 3× mutation (fig. S18, A, B, and E) and from three additional ALS lines from patients with TDP-43^N390D^, TDP-43^M337V^, and c9orf72 hexanucleotide repeat expansion (HRE) mutations (fig. S18, C, D, and F). Thus, we showed how neurons from various patients with PD (PARK2 and SNCA 3× mutations) or ALS (TDP-43^N390D^, TDP-43^M337V^, and c9orf72 mutations) display lower intracellular ATP levels than healthy neurons, resulting in a significant decrease in their axonal cytosol solubility.

### NMN chronic treatment restores intracellular ATP levels and axonal cytosol fluid phase in PD hGNs and ALS hMNs, and reduces TDP-43 pathological aggregation in ALS hMNs

**Fig. 7.**
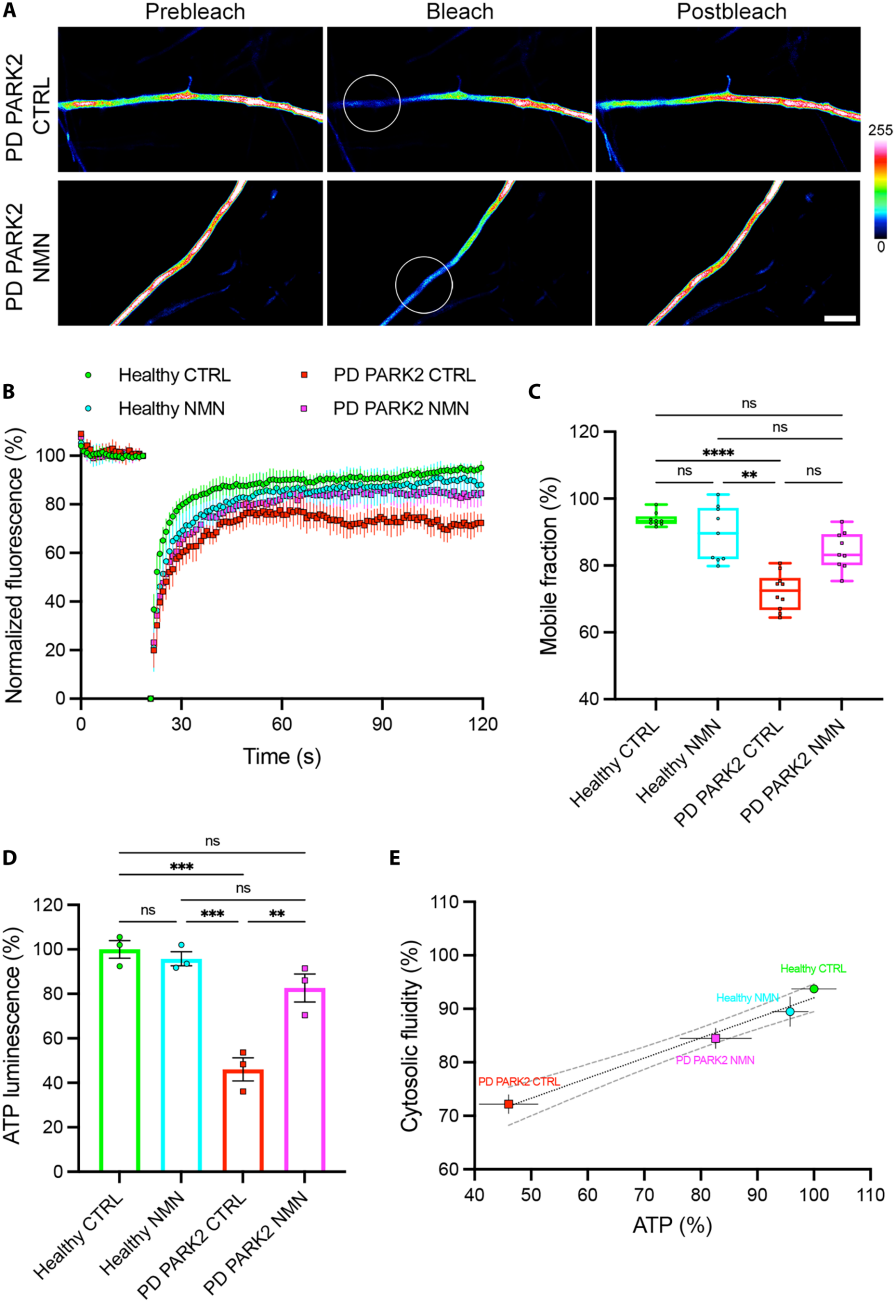
NMN chronic treatment rescues axonal cytosol fluidity and intracellular levels of adenosine triphosphate (ATP) in PD PARK2 iPSC-derived neurons. (**A**) Untreated and NMN-treated axons from PD PARK2 hGNs expressing cGFP (pseudocolor). FRAP ROI before bleach, during bleach, and after recovery. Scale bar, 2 μm; color bar, cGFP fluorescence intensity. (**B**) Fluorescence intensity recovery profiles from healthy, NMN-treated healthy, PD PARK2, and NMN-treated PD PARK2 hGNs. Means ± 95% CI. (**C**) Mobile cGFP fraction representing cytosolic fluidity estimated from the past 20 s of the fluorescence intensity profile in untreated hGNs (*n* = 10 cells from three independent experiments) and NMN-treated healthy hGNs (*n* = 9 cells from three independent experiments) and in untreated PD PARK2 hGNs (*n* = 10 cells from three independent experiments) and NMN-treated PD PARK2 hGNs (*n* = 9 cells from 3 independent experiments). Box plot with median and minimum/maximum whiskers. Kruskal-Wallis nonparametric test with Dunn’s correction. **P = 0.0014 and ****P < 0.0001. (**D**) In vitro bioluminescence measurement of intracellular ATP in untreated and NMN-treated healthy hGNs and in untreated and NMN-treated PD PARK2 hGNs. Means ± SEM, one-way ANOVA with Tukey’s correction. **P = 0.0029 and ***P = 0.0002 and 0.0004. (**E**) Correlation between intracellular level of ATP and cytosolic fluidity of healthy and NMN-treated healthy hGNs and of PD PARK2 and NMN-treated PD PARK2 hGNs. Means ± SEM with simple linear regression (dotted line; *r*^2^ = 0.96) and 95% CI (dashed lines).

**Fig. 8.**
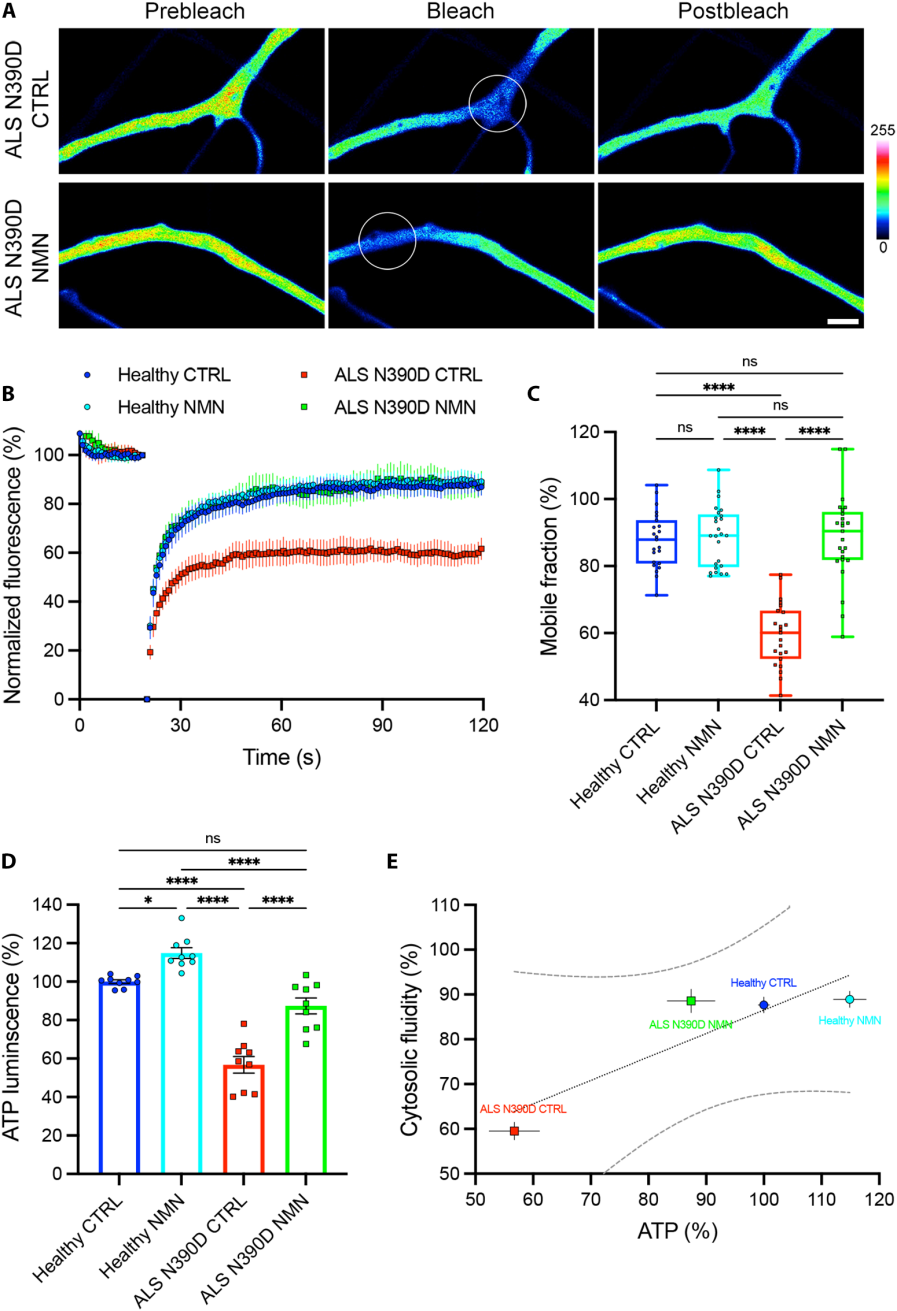
NMN chronic treatment rescues axonal cytosol fluidity and intracellular level of ATP in amyotrophic lateral sclerosis (ALS) TDP-43^N390D^ hMNs. (**A**) Untreated and NMN-treated axons from ALS N390D hMNs expressing cGFP (pseudocolor). FRAP ROI before bleach, during bleach, and after recovery. Scale bar, 2 μm; color bar, cGFP fluorescence intensity. (**B**) Fluorescence intensity recovery profiles of healthy, NMN-treated healthy hMNs, and ALS N390D or NMN-treated ALS N390D hMNs. Means ± 95% CI. (**C**) Mobile cGFP fraction representing cytosolic fluidity estimated from the past 20 s of the fluorescence intensity profile of untreated (*n* = 23 cells from three independent experiments) and NMN-treated (*n* = 25 cells from three independent experiments) healthy hMNs and untreated (*n* = 23 cells from three independent experiments) and NMN-treated (*n* = 25 cells from 3 independent experiments) ALS N390D hMNs. Box plot with median and minimum/maximum whiskers. Kruskal-Wallis nonparametric test with Dunn’s correction. ****P < 0.0001. (**D**) In vitro bioluminescence measurement of intracellular ATP of untreated and NMN-treated healthy hMNs and untreated and NMN-treated ALS N390D hMNs (nine samples from three independent experiments). Means ± SEM, one-way ANOVA with Tukey’s correction. *P = 0.0180 and ****P < 0.0001. (**E**) Correlation between intracellular level of ATP and cytosolic fluidity of healthy and NMN-treated healthy hMNs and ALS N390D and NMN-treated ALS N390D hMNs. Means ± SEM with simple linear regression (dotted line; *r*^2^ = 0.81) and 95% CI (dashed lines).

NMN is a known activator of the NAD^+^ pathway and has already been reported to be an antiaging and neuroprotectant agent (*41*, *59*). We performed chronic treatment with NMN on PD hGNs and ALS hMNs for 1 week before cGFP FRAP analysis and ATP measurements. In the presence of NMN, axons from PARK2 PD and TDP-43^N390D^ ALS neurons displayed a 1.6-fold increase in cGFP fluorescence recovery compared to untreated controls (Figs. 7, A and B, and 8, A and B) and a significant increase in their mobile fraction (Figs. 7C and 8C). In addition, the intracellular level of ATP also increased by 1.4-fold in NMN treated PARK2 and TDP-43^N390D^ neuronal cultures compared to untreated cultures (Figs. 7D and 8D). NMN chronic treatment was able to restore axonal fluid phase and intracellular levels of ATP in PARK2 and TDP-43^N390D^ neurons to the levels observed in healthy neurons, while it had no significant effect on healthy neurons (Figs. 7, B to D, and 8, B to D). We observed a substantial correlation between the intracellular level of ATP and the axonal fluid phase in hGNs and hMNs treated with NMN (Figs. 7E and 8E). We also confirmed the rescue of axoplasmic fluidity and ATP levels upon NMN treatment in the SNCA 3× lines from a patient with PD, as well as in the additional TDP-43^N390D^, TDP-43^M337V^, and c9orf72 lines from patients with ALS (fig. S19, A to F).

**Fig. 9.**
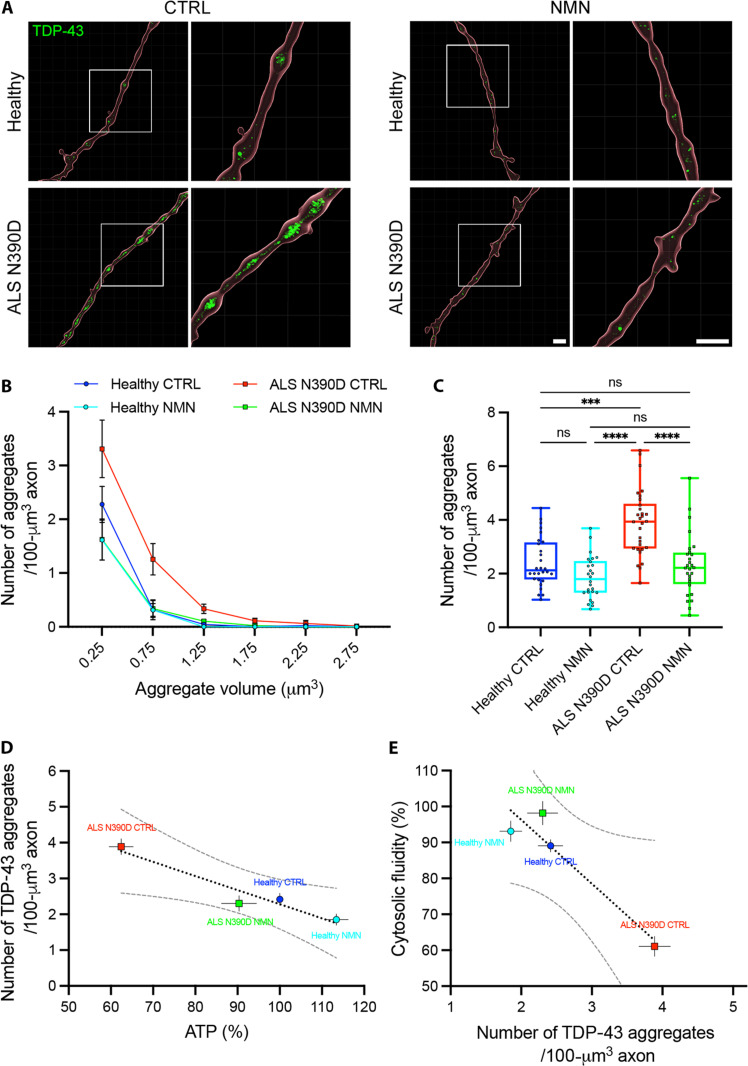
NMN chronic treatment reduces pathological aggregation of TDP-43 in axons of hMNs derived from an amyotrophic lateral sclerosis (ALS) patient with TDP-43^N390D^ mutation. (**A**) Three-dimensional (3D) images of axons from untreated and NMN-treated healthy and ALS N390D hMNs labeled with TDP-43 (green) and β_3_-tubulin (red; surface rendering to delineate axon contour). Scale bars, 5 μm. (**B**) Frequency distribution of TDP-43 aggregate size in axons from untreated and NMN-treated healthy and ALS N390D hMNs. Means ± SEM. (**C**) Average number of TDP-43 aggregates in axons from untreated healthy (*n* = 28 from three independent experiments) and ALS N390D (*n* = 30 from three independent experiments) hMNs and from NMN-treated healthy (*n* = 26 from three independent experiments) and ALS N390D (*n* = 28 from three independent experiments) hMNs. Box plot with median and minimum/maximum whiskers. Kruskal-Wallis nonparametric test with Dunn’s correction for multiple comparison. ***P = 0.0002 and ****P < 0.0001. (**D**) Correlation between intracellular level of adenosine triphosphate (ATP) and number of TDP-43 aggregates in untreated or NMN-treated healthy hMNs and untreated and NMN-treated ALS N390D hMNs. Means ± SEM with simple linear regression (dotted line; *r*^2^ = 0.89) and 95% CI (dashed lines). (**E**) Correlation between TDP-43 aggregation and cytosolic fluidity in axons from untreated or NMN-treated healthy hMNs and untreated and NMN-treated ALS N390D hMNs. Simple linear regression (dotted line; *r*^2^ = 0.91) and 95% CI (dashed line).

Next, we aimed to correlate the data on axoplasmic fluidity with the aggregation of individual proteins, such as TDP-43, whose abnormal LPS has been detected in neurodegenerative diseases. Although we did not detect any accumulation of TDP-43 in axons of PARK2 PD neurons, we showed that NMN chronic treatment significantly reduced pathological aggregation of TDP-43 in axons from TDP-43^N390D^ ALS hMNs (Fig. 9A). In the absence of NMN treatment, axons from TDP-43^N390D^ ALS hMNs displayed consistently larger and more abundant (~2-fold) TDP-43 aggregates than axons from healthy hMNs (Fig. 9, B and C), which were reduced upon NMN treatment compared to untreated axons. NMN treatment, however, did not significantly alter the number of TDP-43 aggregates in healthy hMNs (Fig. 9C). We also observed a robust correlation between the intracellular levels of ATP and the number of TDP-43 aggregates (Fig. 9D), as well as between TDP-43 aggregation and cytosolic fluid phase in those motor neurons (Fig. 9E). Thus, our results strongly suggest a beneficial role of NMN in the restoration of the physiological levels of intracellular ATP and LPS, and in the solubilization of pathological TDP-43 aggregates in motor neurons derived from ALS patients with the TDP-43^N390D^ mutation.

**Fig. 10.**
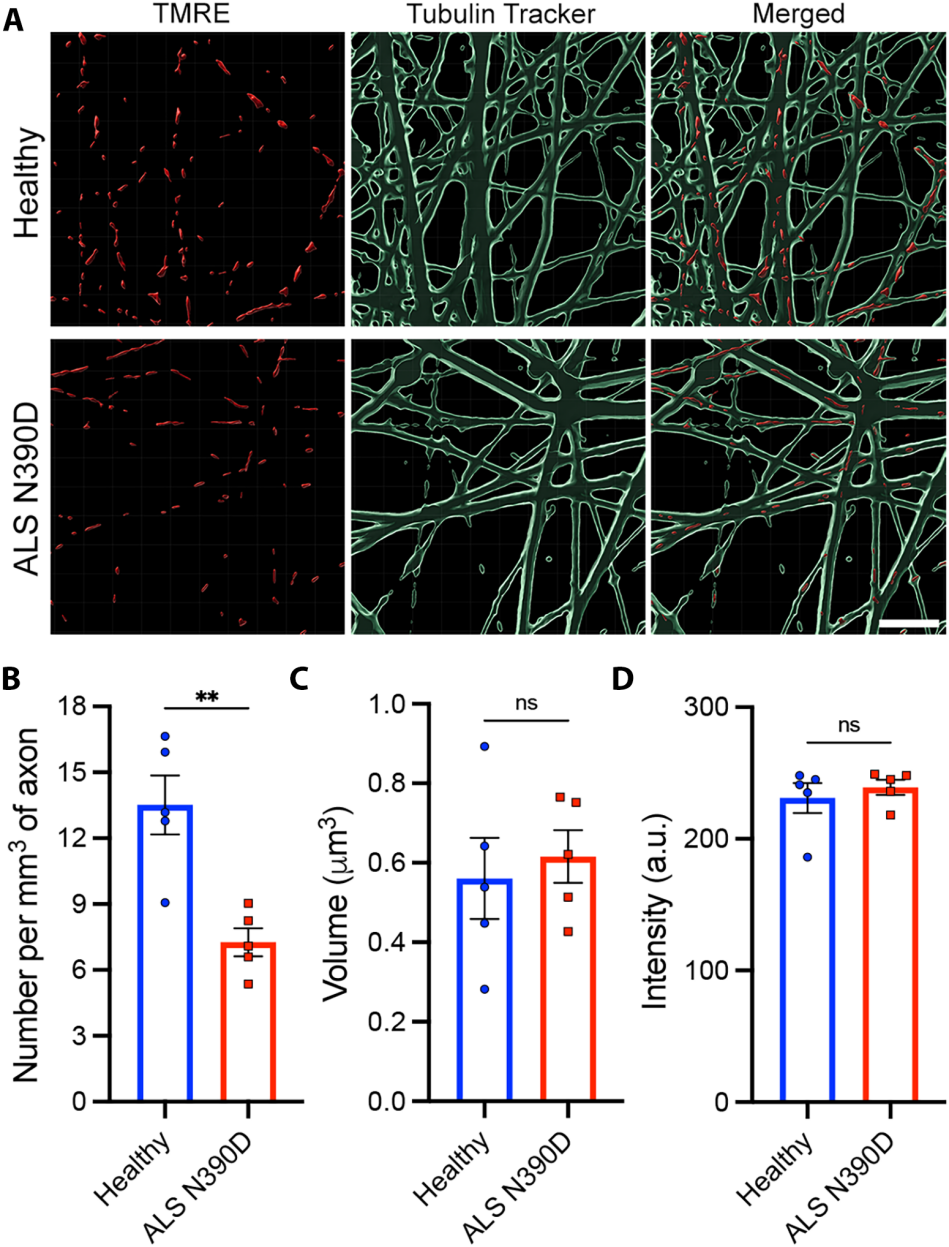
Loss of active mitochondria in axons of hMNs derived from an amyotrophic lateral sclerosis (ALS) patient with TDP-43^N390D^ mutation. (**A**) 3D images of active mitochondria (TMRE; red) from healthy (top) and ALS N390D (bottom) hMNs. The axonal network is labeled with Tubulin Tracker (green). Scale bar, 5 μm. (**B**) Average number of active mitochondria per cubic millimeter of axon from healthy and ALS N390D hMNs. (**C**) Average volume of active mitochondria in axons from healthy and ALS N390D hMNs. (**D**) Average fluorescence intensity of active mitochondria in axons from healthy and ALS N390D hMNs. Means ± SEM (*n* = 5 independent experiments), unpaired Student’s *t* test. **P = 0.0062 (B). a.u., arbitrary units.

We further checked whether this accumulation of TDP-43 in axons was dependent on the presence or absence of active mitochondria. We quantified the number and morphology of TMRE-labeled mitochondria in axons of TDP-43^N390D^ hMNs and observed a significant decrease in the number of active mitochondria compared to healthy axons (Fig. 10, A and B). However, no significant changes in mitochondrial volume or signal intensity were observed (Fig. 10, C and D). These data suggest that the pathological aggregation of TDP-43 observed in axons from a ALS patient with TDP-43^N390D^ mutation might result from the loss of active mitochondria and local reduction in the levels of ATP.

Last, to understand how NMN treatment contributes to restoring ATP levels, we measured intracellular levels of both ATP and NAD/reduced form of NAD^+^ (NADH) in healthy and ALS TDP-43^N390D^ motor neurons. ALS TDP-43^N390D^ neurons showed a significant decrease in intracellular ATP and NAD/NADH compared to healthy neurons, which could be rescued by NMN treatment (fig. S20). Blocking glycolysis with 2-DG, however, reduced the level of ATP recovered from NMN treatment in ALS TDP-43^N390D^ neurons (fig. S20). These results suggest a possible connection between the decrease in intracellular levels of NAD/NADH in ALS TDP-43^N390D^ and the impairment of ATP synthesis from both mitochondrial activity and glycolysis.

Together, our data on PD and ALS neurons, which are summarized in table S1, demonstrate that ATP plays a critical role in the regulation of axoplasmic viscosity and that the local production of ATP is paramount to reduce the pathological protein aggregations observed in neurodegenerative diseases such as PD and ALS. The importance of ATP-dependent regulation of axoplasmic fluidity is reinforced by the finding that the data obtained from our FRAP analysis on axoplasmic viscosity and the measurements of ATP levels from healthy, PD, and ALS iPSC lines could discriminate and sort healthy versus disease neurons using *K*-means cluster analysis (fig. S21).

## DISCUSSION

Accurate measurement of fluorescently tagged protein diffusion, or viscoelasticity in small synaptic boutons, often requires complex modeling of FRAP data, which considers the morphological constraint and crowdedness of the synapse, to accurately describe different modes of diffusion (*47*). Nonetheless, we chose to estimate axoplasmic viscosity based on the analysis of cGFP diffusion by FRAP in mouse giant glutamatergic synapses and in axons of hiPSC-derived neurons. Less than 2% of cGFP has been described to localize to organelles (*47*), with the vast majority remaining highly soluble and free to diffuse in the cytoplasm, while the morphological constraints of giant calyceal synapses are significantly lower than conventional small synaptic boutons (*46*). Our microrheology experiment on extracted cytosol also confirmed the validity of our FRAP measurements (fig. S17). In addition, we excluded the possibility that the change we observed in axoplasmic viscoelasticity upon ATP reduction resulted from cytotoxicity or pH shift (figs. S3 and S7), as well as from a putative reduction in enzymatic activity linked to the energetic properties of ATP, as shown by the absence of any impairments in the active transport of SVs between calyceal swellings (fig. S6). While we are aware of the limitations of our FRAP analysis, we believe that our experimental approach provides a valid assessment of the hydrotropic function of ATP on the cytosolic viscoadaptation in living neurons, as previously reported in yeast (*49*).

We investigated the hydrotropic activity of ATP on the axoplasmic viscoadaptation in rodent giant synapses and showed how mitochondrial activity can locally modulate ATP concentration, thus promoting or preventing the condensation of the presynaptic cytosol and affecting various components (SVs and AZs) of the presynaptic release machinery. Phase separation of SYN1 or RIM1 and their potential involvement in the regulation of SV pool clustering, or assembly of AZs, have been previously shown in vitro (*5*, *6*). We showed that VGLUT2-containing SVs and RIM1 viscosity are reduced upon mitochondrial inactivation in mCTs (fig. S5). In addition, our data suggest that the presynaptic cytosolic viscosity itself is also reduced when ATP is depleted (Fig. 1). Several proteins present in presynaptic cytosol have recently been reported to potentially undergo LPS (*8*), although their dependence on ATP remains to be investigated. LPS has also been reported for proteins of the postsynaptic compartment; SynGAP and PSD-95, for example, have been proposed to organize the postsynaptic density via phase separation (*60*, *61*). Calcium- and calmodulin-dependent protein kinase II (CamKII), a key component of synaptic homeostasis, was found to phase-separate and promote the condensation of AMPA receptors (AMPARs) and neuroligin to cross-link postsynaptic density proteins (*62*). It is likely that both ATP- and CamKII-mediated phase separations might act in synergy to modulate transsynaptic organization of SVs, AZs, and AMPARs, and these mechanisms need to be further investigated.

In addition, our in vitro data demonstrate the ATP dependency of LPS for a variety of proteins involved in neurodegeneration (Fig. 3 and fig. S10). We observed that SNCA^A53T^, Tau, and PARK2 require higher ATP concentration (~4 to 6 mM) to decondense, while SYN1 and TDP-43 only require ~2 to 3 mM. The role of ATP in regulating LPS has also been recently reported in vitro for FUS (*63*–*65*) and TDP-43 (*66*). In agreement with our current observations, TDP-43 was shown to condense below 1.5 mM and decondense at around 2.2 mM ATP (*66*). We also show that mitochondrial inhibitor FCCP treatment leads to ~3-fold reduction in intracellular concentration of ATP compared to untreated terminals (Figs. 1 and 2). Assuming that the intracellular concentration of ATP in mammalian cells is around 4 to 8 mM (*67*), we estimated a resulting ATP concentration after FCCP treatment below 3 mM, which would predict a perturbation of SNCA^A53T^, Tau, and Parkin LPS according to our in vitro measurements. Although we did not monitor LPS for individual proteins, we observed a significant condensation of the presynaptic cytosol with the formation of larger cytosolic aggregates concentrating cGFP in FCCP-treated calyceal terminals. Accordingly, the delivery of 4 to 8 mM ATP to calyceal terminals was able to reduce cytosolic condensates even in the absence of mitochondrial activity (Fig. 2). Thus, our current work, as well as the previous report (*27*), suggests a significant role for intracellular ATP to act as a key regulator of LPS and protein aggregation in neurons and in other cell types. SNCA^A53T^ mutant protein phase-separated in some of the largest condensates among all the proteins we tested. These condensates appeared to aggregate together into elongated fibrils (Fig. 3), closely resembling SNCA protofibrils observed during the early onset of PD (*54*, *68*). These observations suggest that the formation of pathological SNCA protofibrils might be initiated by LPS. It has recently been reported that SNCA and Tau can condense together, with an accumulation of SNCA in Tau droplets (*69*). In addition, our in vitro data also demonstrate that SNCA^A53T^ and Tau aggregates can decondense within higher range of ATP concentration (Fig. 3). We showed that TDP-43, a well-characterized protein implicated in ALS and known to aggregate (*70*, *71*), also phase-separates in an ATP-dependent manner but forms smaller condensates and requires a lower concentration of ATP to decondense (Fig. 3) compared to SNCA^A53T^ or Tau. Thus, pathological protein aggregation of SNCA, Tau, and TDP-43 appears to be regulated in part by the hydrotropic activity of ATP, with different thresholds or sensitivity to ATP for their condensation/decondensation.

Last, axons of neurons differentiated from iPSCs of patients with PD and ALS displayed a significant decrease in their intracellular ATP concentration (3.2- and 1.4-fold in PD and ALS, respectively) concomitant with an increase in the condensation of their axoplasm (1.5- and 1.4-fold in PD and ALS, respectively; Figs. 5 and 6). Although PD and ALS neurons show a similar reduction in axoplasmic fluid phase, the intracellular level of ATP in PD neurons appear to be two times lower than that in ALS neurons. This discrepancy might be related to the different sensitivity of SNCA and TDP-43 previously mentioned. However, our data demonstrate that ATP-dependent LPS is a common molecular mechanism shared in central and peripheral neuropathologies such as PD and ALS, in which protein aggregation is a hallmark of the disease (*17*). The reduction in intracellular ATP concentration observed in our PD and ALS neurons can also be explained by numerous mitochondrial dysfunctions reported in PD, AD, and ALS (*35*, *37*, *39*). In addition, synaptic dysfunctions are also associated with neuropathologies such as PD, AD, and ALS (*10*, *11*), ultimately leading to synapse and axon degeneration before neuronal cell death (*72*, *73*). Tau condensates have also been shown to recruit and increase tubulin nucleation and microtubule (MT) polymerization (*74*). Consequently, MT overpolymerization has been recently reported to impair neurotransmission in a mouse giant synapse model of AD, where the injection of Tau in terminals increased MT polymerization, sequestering Dynamin-1 on MTs and impairing synaptic transmission (*75*).

Our findings reveal the importance of ATP-dependent viscoadaptation in PD and ALS pathologies (Figs. 5 and 6 and table S1), as well as the rescue of normal axoplasmic viscosity and decondensation of pathological aggregates by chronic NMN treatment in PD and ALS neurons (Figs. 7 and 8 and table S1). NMN has been previously shown to enhance energy production and neuronal survival (*41*, *42*, *44*) and to mitigate age-associated decline in rodents (*59*). We also report that NMN treatment has the potential to reduce the pathological aggregation of TDP-43 in axons of ALS TDP-43^N390D^ mutant neurons (Fig. 9).

Whether ATP-dependent viscoadaptation of the axonal cytosol is a common molecular mechanism in all familial or sporadic neurodegenerative diseases remains to be investigated. Nonetheless, we believe that intracellular ATP levels are pivotal to the regulation of axoplasmic viscosity and maintain cellular homeostasis and function. Our current work expands the field of research on the role of ATP in neurodegenerative diseases, which, up to recently, focused mainly on its energetic activity (*76*, *77*). Future research and development to improve NAD/NADH-dependent pathways and ATP production might be beneficial for the development of future prophylactics or therapeutics to prevent or alleviate protein aggregation in neurodegenerative diseases. Collectively, our data support the role of ATP as a cellular hydrotrope and that ATP-dependent protein condensation/decondensation of neuronal proteins via LPS plays a critical role in the cytosolic viscoadaption of axons and synapses in mammalian neurons during health and disease.

### Limitations of the study

Although our data suggest the possible role of ATP in the regulation of axoplasmic viscosity and protein aggregation, arguing in support of its hydrotropic activity in cellular context, we do not exclude the possibility that the binding and canonical enzymatic activity of ATP also contribute to the regulation of protein solubility, as previously reported (*78*).

Direct delivery of ATP into cells also remains technically challenging. We used chitosan-mediated delivery of ATP in our synapse culture model, but this method induced temporary permeabilization of the plasma membrane and, thus, could potentially affect cytosol viscosity. An alternative approach would have been the use of ATPsomes (liposome) to deliver ATP into the cells. This approach, however, does not allow for a fine control of the concentration of ATP delivered to cells. Thus, we preferred to use a more physiological approach to boost ATP synthesis by NMN chronic treatment. We showed in mouse the calyx of Held synapse model that the production of ATP upon glycolysis inhibition by 2-DG did not contribute to the regulation of the synaptic cytosolic viscosity, as expected since synaptic transmission at the calyx of Held is mainly sustained by mitochondrial respiration (*79*). However, we observed a significant reduction in ATP production in hMN axons after NMN treatment with the glycolysis inhibitor 2-DG. NAD/NADH levels appeared to be reduced in ALS motor neurons, and, as NAD/NADH plays a role in glycolysis, mitochondrial oxidative phosphorylation, and the tricarboxylic acid cycle, the coupling or uncoupling of these different pathways needs to be further investigated to strengthen our understanding of their respective role in ATP production and viscoadaption in neurons.

In addition, our preliminary in cellulo results on NMN treatment advocate for further investigation of the potential prophylactic or therapeutic role of NAD^+^ activator in neuronal proteinopathies such as PD or ALS. The safety of NMN oral administration has already been tested in human clinical trials without any significant or serious adverse effects (*80*, *81*), and other NAD^+^ precursors, such as NR (*43*) or nicotine (*82*), have also been reported to improve age-related symptoms. Whether NMN treatment could prevent pathological protein aggregation before or during the onset of ALS remains to be further explored and confirmed in other iPSC lines and animal models before potential human trials. Because of the complexity and multifactorial aspects of neurodegenerative diseases such as PD or ALS, targeting ATP synthesis only might not be sufficient; however, in combination with other therapeutic approaches, it might contribute to the development of novel and more effective treatments.

## MATERIALS AND METHODS

### Reagents and chemicals

Mitochondrial membrane potential assay kit (#ab287864) containing TMRE and uncoupler of mitochondrial oxidative phosphorylation FCCP was purchased from Abcam. Mitochondrial complex 1 inhibitor rotenone (#3616) and ATP synthase inhibitor oligomycin A (#4110) were purchased from Tocris. Glycolysis inhibitor 2-DG (#D8375), ATP (#A6419), and β-NMN (#N3501) were purchased from Sigma-Aldrich. Anti–TDP-43 antibody (#10782-2-AP) was purchased from Proteintech; anti–β_3_-tubulin antibody (#302304) and anti-SYN1 antibody (#106103) were purchased from Synaptic System GmbH. NucBlue for live (#R37605) or fixed (#R37606) cells and Alexa Fluor 488–, Alexa Fluor 568–, and Alexa Fluor 647–conjugated secondary antibodies were purchased from Thermo Fisher Scientific. Goat anti-rabbit horseradish peroxidase (HRP)–conjugated secondary antibody (#ab6721) was purchased from Abcam, and enhanced chemiluminescence (ECL) start detection reagents kit (#RPN3243) was purchased from Cytiva.

### Primary dissociated culture of mouse calyceal synapses

Pregnant mice were purchased from Charles River Laboratories Japan, and dissociated neuron culture from brainstem was performed as previously described (*45*). Briefly, mouse brains from postnatal day 1 pups were extracted, and the cochlear nucleus (CN) and the medial nucleus of the trapezoid body (MNTBs) regions were microdissected and stored separately in ice-cold Hanks’ balanced salt solution. CN and MNTB regions were dissociated using nerve cell dissociation kit (Sumitomo Bakelite) according to the manufacturer’s instructions. Dissociated neurons were then plated at an equal ratio of CN and MNTB neurons to a final density of 200,000 cells per 35-mm culture dish (#80131, ibidi), previously coated with poly-d-lysine (100 mg/ml; #A-003-E, Sigma-Aldrich), and cultured in astroglial-conditioned nerve cell culture medium (Sumitomo Bakelite) supplemented with nerve growth factor (100 ng/ml; #13257-019, Thermo Fisher Scientific), human brain-derived neurotrophic factor (hBDNF; 25 ng/ml; #450-02, PeproTech), human fibroblast growth factor (5 ng/ml; #100-18B, PeproTech), human neurotrophin-3 (hNT-3; 50 ng/ml; #450-03, PeproTech), and 20 mM KCl. At day in vitro 8 (DIV8), 5 mM arabinofuranosylcytosine (AraC; #C1768, Sigma-Aldrich) was added to the medium to inhibit cell proliferation. Medium without AraC was exchanged every 4 days throughout the culture and until experiments.

### Culture of hiPSC-derived glutamatergic neurons

Plasmids containing neurogenin 2 expression cassettes were a gift from the T. Südhof laboratory. Lentiviral particles were prepared in Lenti-X 293T cells (#632180, Takara) using the Lenti-X Packaging Single Shots (vesicular stomatitis virus glycoprotein) (#631275, Takara) according to the manufacturer’s instructions. Particles were concentrated by centrifugation at 100,000*g* for 2 hours and resuspended in Neurobasal Plus medium and kept at −80°C until use. hiPSC lines were derived from a healthy individual (#HPS0077) or from a patient with PD and PARK2 mutation (#HPS0097) from Riken Cell Bank (Japan) and SNCA 3× mutation (#EDi001-A) from European Bank for Induced Pluripotent Stem Cells (Germany). The PARK2 (*36*, *83*) and SNCA (*84*) lines used in this study have been well characterized for their mitochondrial dysfunction and SNCA aggregation. Differentiation into neurons was based on lentiviral delivery and tetracycline-inducible expression of neurogenin 2 transcription factor and puromycin selection, as previously described (*85*). Briefly, iPSCs were maintained in StemFit medium (#RCAK02N, Ajinomoto Co., Japan). On day 1 of differentiation, cells were plated in 35-mm culture dishes. On day 2, lentiviral particles were added in differentiation medium containing Dulbecco’s modified Eagle’s medium (DMEM)/F12 medium supplemented with N2 (#17502048, Thermo Fisher Scientific), nonessential amino acid (#11140050, Thermo Fisher Scientific), and doxycycline (2 μg/ml; #24390-14-5, Sigma-Aldrich). On day 3, differentiation medium was changed additionally containing puromycin (1 μg/ml; #P8833, Sigma-Aldrich). On day 5, the cells were replated on 35-mm Ibidi dishes in maintenance medium containing Neurobasal Plus and B27 Plus (#A3582901 and #A3653401, Thermo Fisher Scientific) supplemented with BDNF (10 ng/ml) and hNT-3 (10 ng/nl), laminin (0.5 μg/ml; #23017015, Thermo Fisher Scientific), and doxycycline. On the fifth day after replating, human astrocytes (#1800, ScienCell) and AraC (5 μM) were added to the culture during a half-medium change. Half of the culture medium was replaced with fresh medium every 5 days until the day of the experiment. Doxycycline was kept in the medium throughout the whole culture duration.

### Culture of hiPSC-derived motor neurons

hiPSC control (CS9LL4iCTR-nxx and CS2PFYiCTR-nxx) and ALS (TDP43^N390D^ mutation, CS5ZLDiALS-nxx and CS1UWUiALS-nxx; TDP43^M337V^ mutation, CS3MG8iALS-nxx; c9orf72 HRE mutation, CS29iALS-C9nxx) lines were purchased from Cedars-Sinai Biomanufacturing Center (USA). The TDP-43 lines used in this study harbors a mutation (N390D) found in TDP-43–linked sporadic cases of ALS, which maps in the C-terminal domain and has been characterized for its pathogenic effect. Thus, data collected with the TDP-43^N390D^ mutants will likely be translatable to most common ALS-linked TDP-43 mutations affecting its aggregation propensity. In addition, we selected another cell line with a different mutation of TDP-43^M337V^, which also maps in the C-terminal, a glycine-rich region, key to the regulation of TDP-43 solubility and folding, together with a non–TDP-43 mutant line, which harbors a mutation in the c9orf72 gene. hiPSCs were cultured in StemFit medium, and motor neuron differentiation was performed as described previously (*86*). Briefly, iPSCs were grown in a 100-mm cell culture dish until confluent, harvested, and placed into non–cell culture–treated dishes. To obtain embryoid bodies (EBs), for the first 2 days, cells were allowed to grow in suspension in StemFit medium supplemented with basic fibroblast growth factor (20 ng/ml; #100-18B, PeproTech) and 20 μM Rho-associated kinase (ROCK) inhibitor Y27632 (#SCM075, Chemicon International) or RevitaCell (#A2644501, Invitrogen) to enhance cell survival. On the third day, neuralization was induced by the addition of 10 μM SB431542 (#MBS808210, Biosource Filgen) and 0.2 μM LDN193189 (#MBS385030, Biosource Filgen) to the cultures. On the fourth day, EBs were switched to neural induction medium (DMEM/F12, #11320033, Thermo Fisher Scientific), GlutaMAX (#35050061, Thermo Fisher Scientific), Primocin (#ant-pm-1, Invivogen), 0.1 mM nonessential amino acids (#11140050, Invitrogen), heparin (2 μg/ml; #H3149, Sigma-Aldrich), and 1% N2 supplement (#17502048, Thermo Fisher Scientific), supplemented with 20 μM ROCK inhibitor, ascorbic acid (0.4 μg/ml; #A4403, Sigma-Aldrich), 1 μM retinoic acid (#R2625, Sigma-Aldrich), and BDNF (10 ng/ml; #450-02, PeproTech). SB431542 and LDN193189 were added until day 7 when cultures were supplemented with 1 μM smoothened agonist (#566660, Calbiochem) and 0.5 μM purmorphamine (#4551/10, Tocris). EBs were grown for an additional 10 days with a medium change every other day. At day 17, EBs were dissociated with 0.05% trypsin and plated on poly-l-lysine/laminin-coated 35-mm glass-bottom culture dishes (#81158, ibidi) at a final density of 300,000 cells per dish. Cells were cultured in neural differentiation medium (Neurobasal medium, #A3582901, Thermo Fisher Scientific), GlutaMAX (#35050061, Thermo Fisher Scientific), Primocin, 0.1 mM nonessential amino acids, and 1% N2 supplement, supplemented with 2% B27 (#A3653401, Thermo Fisher Scientific), 25 μM glutamate (#G5889, Sigma-Aldrich), ascorbic acid (0.4 μg/ml), glial-derived neurotrophic factor (10 ng/ml; #450-10, PeproTech), and ciliary neurotrophic factor (10 ng/ml; #450-13, PeproTech). hMNs were allowed to differentiate and mature for 2 to 3 weeks before experiments.

### cDNA constructs and transfection

cGFP pCAG-AcGFP (*45*), vesicular glutamate transporters pCAG-Venus-VGLUT2 (*46*), pCAG-mTurquoise2-RIM1, GW1-PercevalHR (#49082, Addgene), and pCAG-FusionRed constructs were transfected into mouse neurons by electroporation (Neon Electroporation System, Thermo Fisher Scientific) before plating, as described previously (*46*). Briefly, the pCAG-mTurquoise2-RIM1 was constructed by cloning RIM1 cDNA (#100064122, DNAFORM) and mTurquoise2 cDNA from pmTurquoise2-Tubulin (#36202, Addgene) into pSF-CAG-Kan (#OG505, Oxford Genetics). The pCAG-FusionRed was constructed by inserting FusionRed cDNA from FusionRed-lifeact7 (#54778, Addgene) into pSF-CAG-Kan. hGNs were also transfected with pCAG-AcGFP by electroporation before plating. hMNs were transfected with pCAG-AcGFP using JetPEI (#101000053, Polyplus) between DIV7 to DIV10 or transduced with AAV(PHP.eB)-CAG-GFP (#SL116010, SignaGen Laboratories) on DIV10.

### Live imaging and FRAP experiments

Live imaging of mouse calyceal cultures, hiPSC-derived glutamatergic cultures, and hiPSC-derived motor neuronal cultures expressing various fluorescent proteins (cGFP, Venus-VGLUTs, or mTurquoise-RIM1) were performed on laser confocal microscope LSM780 and LSM900 (Carl Zeiss GmbH) equipped with on-stage incubation chamber P-set2000 (#133-800261, Pecon) at 37°C and 5% CO_2_ and with a plan-apochromat 63× oil-immersion objective [numerical aperture (NA) = 1.4; Carl Zeiss GmbH]. Acquisition settings were adjusted to maintain an acquisition speed of 1 frame/s in the region of interest (initial image parameters: 512 pixel–by–512 pixel resolution, 1.25- to 2.06-μs pixel dwell time, bidirectional scan, and 1 arbitrary unit). Culture medium from mouse calyceal cultures, human glutamatergic cultures and motor neuron cultures was replaced with Tyrode’s solution with sodium bicarbonate (#T2397, Sigma-Aldrich) before live imaging. Mouse calyceal and hGNs were imaged on LSM780, and hMNs were imaged on LSM900. Images were acquired every second for a total of 2 to 3 min, while focus was automatically maintained throughout the acquisition period using DefiniteFocus system (Carl Zeiss GmbH). For FRAP experiments, bleaching (argon or diode laser excitation of 488 nm, 30 or 10 mW, 100% laser power, and 2 or 10 scans at 303 or 53 ms per scan on LSM780 or LSM900, respectively) of the region of interest (3.6 or 3.8 μm in diameter on LSM780 or LSM900, respectively) was started 20 s after the beginning of the acquisition, and fluorescence recovery in the bleached area was monitored for the remaining 2 to 3 min. Standard FRAP analysis was applied by removing background fluorescence and corrected for any fluorescence decay from an unbleached area during the acquisition. All fluorescence recovery intensity profiles were then normalized against the maximum fluorescence intensity on frame 20 (just before the bleach) and the minimum fluorescence intensity at the end of the bleach sequence.

### Measurement of presynaptic ATP in mouse calyceal cultures by live confocal fluorescence microscopy

Local ATP levels were estimated in calyceal terminal overexpressing the fluorescent ATP/ADP sensor PercevalHR, as previously reported (*87*). The fluorescence signal intensity of PercevalHR was quantified in presynaptic regions by live fluorescence confocal microscopy under control condition and in the presence of the mitochondrial activity blocker FCCP.

### Measurement of intracellular ATP and NAD/NADH concentration by bioluminescence in vitro assay

Intracellular ATP and NAD/NADH levels from mouse calyceal cultures, iPSC-derived glutamatergic neurons, and iPSC-derived motor neuron cultures were estimated using CellTiter-Glo 2.0 kit (#G9241, Promega) and NAD/NADH-Glo kit (#G9071, Promega) with GloMax 20/20 luminometer (#E5311, Promega) or VICTOR Nivo multimode plate reader (PerkinElmer), according to the manufacturer’s instructions. Briefly, cells (calyceal cultures, hGNs, or hMNs) were cultured in 24-well plates or 35-mm culture dishes for 2 to 3 weeks. Culture medium was then replaced with Tyrode’s solution, and an equal volume of CellTiter-Glo or NAD/NADH-Glo reagent was added to the well or dish. After incubation for 12 min at room temperature, luminescence was recorded. For comparison, the luminescence value was adjusted to the number of cells in each sample.

### Intracellular delivery of ATP into mouse calyceal cultures

As ATP cannot cross the plasma membrane to penetrate cells, we used chitosan-assisted semipermeabilization of ATP (*88*) to directly deliver ATP into calyceal terminals. A mixture of 32 mM ATP and low–molecular weight deacetylated chitosan (10 mg/ml; #448869, Sigma-Aldrich) was diluted in Tyrode’s solution with sodium bicarbonate to reach various concentrations of ATP (2, 4, and 8 mM) and added to the cell culture for 2 hours at 37°C before live-imaging experiments.

### Measurement of cell viability in mouse calyceal cultures

Cell viability was assessed by Cytopainter Fluorometric Red dye (#ab176744, Abcam) according to the manufacturer’s instructions under control condition or after incubation with 50 μM FCCP for 40 min. Briefly, VCN neurons expressing cGFP were incubated with the dye for 45 min at 37°C and 5% CO_2_. Cells were washed and incubated in Tyrode’s solution with sodium bicarbonate for live imaging on LSM900 confocal microscope. The number of dead cells (Cytopainter-positive) and live cells (GFP-positive) were counted to evaluate the cytotoxicity upon FCCP treatment.

### Measurement of intracellular pH in mouse calyceal cultures

Intracellular pH variations were measured using pHrodo Green AM (#P35373, Thermo Fisher Scientific) according to the manufacturer’s instructions. Briefly, after removing culture medium, pHrodo AM ester staining solution diluted in Tyrode’s solution with sodium bicarbonate was added to the cells and incubated for 30 min at room temperature. Cells were further washed and incubated in Tyrode’s solution containing NucBlue before live imaging on LSM900 confocal microscope. The intensity of pHrodo Green AM was quantified in the cell body of VCN neurons under control condition or after the delivery of 4 mM ATP by chitosan-assisted semipermeabilization. An increase in pHrodo intensity correlates with a drop in cytosolic pH, while a decrease in pHrodo intensity is associated with an elevation of cytosolic pH.

### NMN chronic treatment of mouse calyceal cultures and hMN cultures

hMN cultures at DIV12 to DIV15 were treated with 100 μM NMN for 6 consecutive days and with 1 mM NMN on the last day before imaging.

### Immunofluorescence imaging of TDP-43 in hMNs

After 18 to 21 days in culture, motor neurons were fixed in phosphate-buffered saline (PBS) with 4% paraformaldehyde for 20 min at room temperature and overnight at 4°C. The next day, samples were permeabilized in PBS with 0.1% saponin and blocked in PBS with 5% goat serum and 0.01% saponin for 1 hour at room temperature. Primary antibodies were diluted in PBS with 0.5% goat serum and 0.01% saponin and incubated overnight at 4°C. The next day, samples were rinsed three times in PBS with 0.01% saponin. Alexa Fluor secondary antibodies were diluted in PBS with 0.5% goat serum and 0.01% saponin and incubated for 1 hour at room temperature. Samples were then rinsed three times in PBS with 0.01% saponin and lastly washed in PBS before mounting in ibidi mount solution (#50001, ibidi). Images were acquired on LSM900 (Carl Zeiss GmbH) equipped with a plan-apochromat 63× oil-immersion objective (NA = 1.4; Carl Zeiss GmbH).

### Localization and quantification of active mitochondria in axons

hMNs at DIV18 to DIV21 were first incubated with Tubulin Tracker Green (#T34075, Thermo Fisher Scientific) according to the manufacturer’s instructions. Briefly, cells were labeled with Tubulin Tracker Green and Pluronic F-127 (1:1 volume) solution for 30 min in culture medium at 37°C. Cells were rinsed three times in Tyrode’s solution with sodium bicarbonate and further incubated with TMRE for 15 min before live imaging on LSM900 confocal microscope. Images were acquired with the following parameters: image size of 1024 pixels by 1024 pixels, 1.03-μs pixel dwell time, and 15 z-stacks (240-nm stack). Volume rendering of confocal stacks was performed in Imaris 10 (Bitplane Oxford Instruments) using Tubulin Tracker Green to delineate the axonal network and TMRE to detect mitochondria. The number of mitochondria per cubic millimeter of axon and the mitochondrial volume and signal intensity were determined and compared between healthy and ALS neurons.

### In vitro LPS assay

Condensation and decondensation of purified proteins are commonly observed under phase or differential interference contrast microscopy, where proteins in solution separate to form droplets spontaneously or with the addition of crowding agents such as PEG (*89*). The addition of PEG only facilitates and speeds up the formation of larger protein condensates and is commonly used to generate phase separation diagrams of protein solution within a few minutes after protein and PEG. However, proteins such as SYN1 at 10 μM have been shown to phase-separate in the absence of any crowding agent after 1 hour at room temperature (*5*). Thus, we performed all our observation after 1 to 3 hours at 37°C in the absence of additional crowding agent. Human recombinant full length protein SYN1 (#TP321273) was purchased from OriGene Technologies Inc. Human recombinant full-length proteins SNCA (#pro-393), SNCA^A53T^ mutant (#pro-159), microtubule-associated protein Tau (#pro-295), ubiquitin ligase Parkin (PARK2, #pro-1840), APP N-terminal domain (#pro-1080), TAR DNA binding protein (TDP-43, #pro-2383), and RNA binding protein LIN28 (#pro-743) were purchased from ProSpec. All proteins were diluted at a concentration of 100 μM and stored at −80°C. Frozen stock aliquots were thawed on ice, and proteins were diluted in 20 μl of Tyrode’s solution with sodium bicarbonate at a final working concentration of 10 μM. Protein solutions were then loaded into angiogenesis glass-bottom microwell slides (#81507, ibidi) and incubated for 1 to 3 hour at 37°C. Slides were then transferred into the confocal microscope incubation chamber to maintain a temperature of 37°C and observed with a plan-apochromat 63× (NA = 1.4; Carl Zeiss GmbH) or a plan neofluar 100× (NA = 1.45; Carl Zeiss GmbH) oil immersion objective on LSM780 and LSM900 (Carl Zeiss GmbH). The size and number of condensates were quantified under control condition or in the presence of increasing concentration of ATP (0 to 8 mM).

### Fluorescent labeling of purified proteins and FRAP analysis in vitro

Twenty microliters of SNCA and SNCA^A53T^ protein solution at 1 μg/μl were first microdialyzed against PBS using micro–Tube-O-DIALYZER (molecular weight cutoff, 8 kDa; #786-612 T, G-Biosciences) according to the manufacturer’s instructions. After dialysis, SNCA and SNCA^A53T^ were fluorescently labeled using Alexa Fluor 488 microscale protein labeling kit (#A30006, Thermo Fisher Scientific) according to the manufacturer’s instructions with an optimal dye:protein molar ratio of 10. Fluorescently labeled SNCA and SNCA^A53T^ aliquots (0.4 μg/μl) were frozen and stored at −20°C. Fluorescent proteins were loaded into angiogenesis glass-bottom slides, incubated for 1 hour at 37°C, and imaged as described above.

### SDS gel protein electrophoresis

After boiling for 5 min at 100°C, purified proteins in Laemmli buffer were loaded onto NuPAGE 4 to 12% bis-tris gels (#NP0322BOX, Thermo Fisher Scientific) with PageRuler prestained protein ladder (#26616, Thermo Fisher Scientific) and ran at 120 V in MES SDS running buffer (#B0002, Thermo Fisher Scientific). Gels were stained in colloidal blue solution (#LC6025, Thermo Fisher Scientific) overnight at room temperature. Gels were further destained in Milli-Q water for several hours before imaging on ChemiDoc MP imaging system (Bio-Rad).

### Western blotting of SYN1

SYN1 protein (0.3 μg) was boiled in Laemmli buffer at 100°C for 5 min, loaded onto 4 to 12% bis-tris gels (#NP0322BOX) with PageRuler Plus prestained protein ladder (#26619), run at 170 V for 1 hour, and transferred to nitrocellulose membrane (#LC2000, Thermo Fisher Scientific). Successful transfer was confirmed with 5-min incubation with Ponceau S (#BCL-PSS-01, Beacle Inc.) at room temperature, followed by three washes in tris-buffered saline (TBS)–Tween 20 (0.1%) for 10 min. The blotted membrane was blocked in 5% milk in TBS–Tween 20 (0.1%) for 1 hour at room temperature and then incubated with anti-SYN1 primary antibody (#106103) overnight at 4°C. Afterward, membrane was washed four times with TBS–Tween 20 (0.1%) and incubated with goat anti-rabbit (#ab6721) secondary HRP-conjugated antibody for 1 hour at room temperature. The membrane was lastly washed three times for 30 min with TBS–Tween 20, developed using Cytiva ECL start detection reagents (#RPN3243), and detected using ChemiDoc MP imaging system (Bio-Rad).

### Quantification of cytosolic viscoadaptation by microrheology on purified cytosol from hiPSC-derived motor neurons

At DIV18, around 10^7^ control and FCCP-treated hMNs were resuspended and harvested by centrifugation at 1000*g* for 3 min at room temperature. Supernatants were discarded, and cell pellets were flash frozen in liquid nitrogen and stored at −80°C. The next day, cell pellets were thawed on ice and sonicated for 15 min. Samples were then centrifuged at 10,000*g* for 10 min at 4°C, and supernatants were transferred to a clean tube for a second centrifugation at 16,000*g* for 10 min at 4°C. Supernatants containing purified cytosolic fractions were then frozen and stored at −80°C. After thawing, 20 μl of purified cytosol from control and FCCP-treated cells was loaded onto angiogenesis glass-bottom slide microwells (ibidi). In addition, 20 μl of 15% Ficoll 400 solution was loaded in separate wells for reference. Rhodamine-labeled polystyrene nanoparticles (50 nm in diameter; #PS50-RB-1, NanoCS) were added at a final dilution of 1:2000 per well. Slides were transferred into the LSM900 confocal microscope incubation chamber to maintain a temperature of 37°C and observed with a plan-apochromat 63× (NA = 1.4) oil immersion objective. Time series were acquired with the following parameters: image size of 256 pixels by 256 pixels, 1.33-μs pixel dwell time, 5 z-stacks (200-nm stack), scanning speed of ~200 ms per frame with a total number of 32 images over 60 s. The movements of rhodamine beads were tracked and analyzed using the Brownian motion algorithm in Imaris 10 (Bitplane Oxford Instruments). Mean square displacement (MSD) plots were generated for each track, and the diffusion coefficients (*D*) were extrapolated from the linear fit of the MSD plots under each condition (control, FCCP-treated, or 15% Ficoll).

### Statistical analysis

The investigators were not blinded during data collection. No statistical methods were used to predetermine sample sizes. All datasets were screened for outliers using ROUT (*Q* = 1%) outliers identification test and compared in Prism 10 (GraphPad Software Inc.). *K*-means cluster analysis was performed in DATAtab (DATAtab e.U. Graz, Austria; https://datatab.net). The specific statistical tests used to compare datasets are reported in the corresponding figure legends. The total number of samples *n* was pooled from at least three independent experiments. Statistical significance (*) was assumed when *P* ≤ 0.05. Exact sample sizes and *P* values are presented in figure legends.

### Image and figure preparation

All confocal images were acquired with Zen black and Zen blue software version 3 (Zeiss). Median filter was applied on confocal images, and filtered images were exported in Tagged Image File Format (TIFF) image format. Three-dimensional (3D) surface rendering and measurements were performed in Imaris (Bitplane Oxford Instruments), and 3D images were exported in TIFF format. TIFF files were further processed in Photoshop (Adobe) to create final figures.

### Ethics statement

All experiments have been performed in accordance with the regulations of Okinawa Institute of Science and Technology (OIST) animal care and use committee (protocol #2015-128). OIST animal facilities and animal care and use program are accredited by the Association for Assessment and Accreditation of Laboratory Animal Care International (reference #1551). DNA recombinant experiments were approved by OIST biosafety committee (protocols #RDE-2020-020-3 and #RDE-2015-001-6).
