# Supplementary material for: Loss of intracellular ATP affects axoplasmic viscosity and pathological protein aggregation in mammalian neurons
Source: Sci Adv. 2025 Apr 23;11(17):eadq6077. doi: 10.1126/sciadv.adq6077 (PMC12017319; doi:10.1126/sciadv.adq6077)
Supplement: Supplementary file 1 — Figs. S1 to S21 Table S1 [file sciadv.adq6077_sm.pdf]

Science Advances
AAAS

Supplementary Materials for

Loss of intracellular ATP affects axoplasmic viscosity and pathological protein aggregation in mammalian neurons

Laurent Guillaud *et al.*

Corresponding author: Laurent Guillaud, laurent.guillaud@oist.jp; Marco Terenzio, marco.terenzio@oist.jp

Sci. Adv. **11**, eadq6077 (2025)
DOI: 10.1126/sciadv.adq6077

This PDF file includes:

Figs. S1 to S21
Table S1

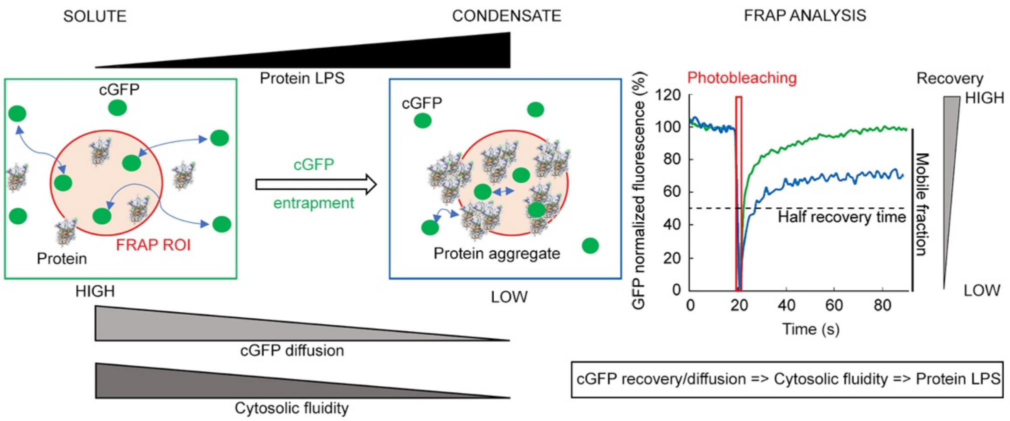

Fig. S1: Assessment of protein LPS by FRAP analysis of cytosolic GFP.
In normal conditions, with proteins in solute form and high cytosol fluidity, cGFP molecules (green dots) can easily diffuse into the bleached area (red ROI), allowing for high fluorescence recovery. Upon LPS condensation, protein aggregates reduce cytosol fluidity, thus impairing the diffusion of cGFP molecules into the bleached area, resulting in slower fluorescence recovery. Quantification of cGFP recovery/diffusion provides an accurate estimate of the global cytosolic viscosity and thus the global level of protein condensation via LPS.

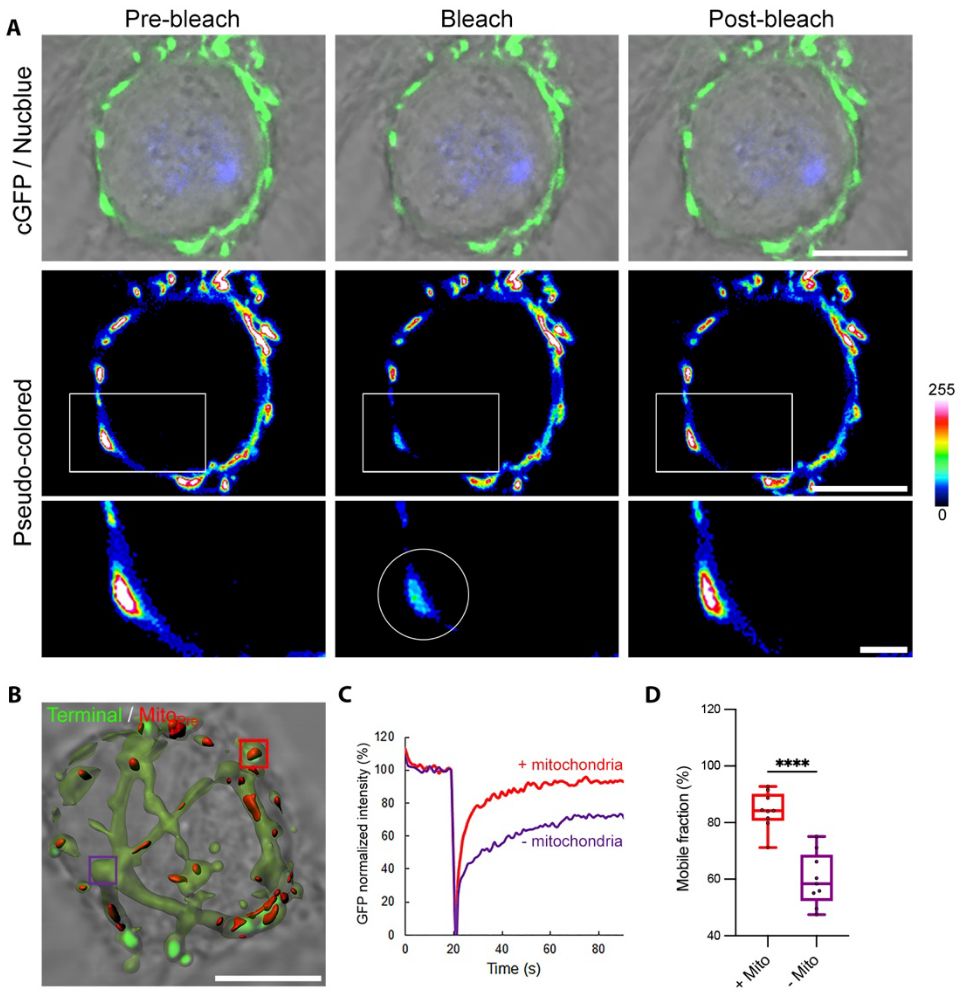

Fig. S2: Analysis of presynaptic cytosol fluidity by FRAP in mCTs.

(**A**) Live confocal images of mCTs expressing cGFP (upper panels) and corresponding pseudo-colored images (lower panels) in culture for 18-21 days. FRAP was performed on ROI containing at least one calyceal swelling (white circle). Sequential images show ROI before bleach (left panels), during bleach (middle panels) and after recovery (right panels), scale bars = 10 µm and 2 µm, color bar = cGFP fluorescence intensity. (**B**) 3D surface rendering of calyceal terminal expressing cGFP (green) and labeled with TMRE showing only presynaptic mitochondria (red). Colored squares represent the area where cGFP FRAP was performed. (**C**) cGFP fluorescence recovery profiles in presynaptic regions with active mitochondria (red) or without active mitochondria (purple), scale bar = 10 mm. (**D**) Quantification of the mobile fraction in presynaptic regions with (red) and without (purple) active mitochondria (n = 9 terminals from 3 independent experiments). Data are represented as box plot with median and minimum/maximum whiskers. Statistical analysis was performed with Mann-Whitney nonparametric test. ****: $p < 0.0001$.

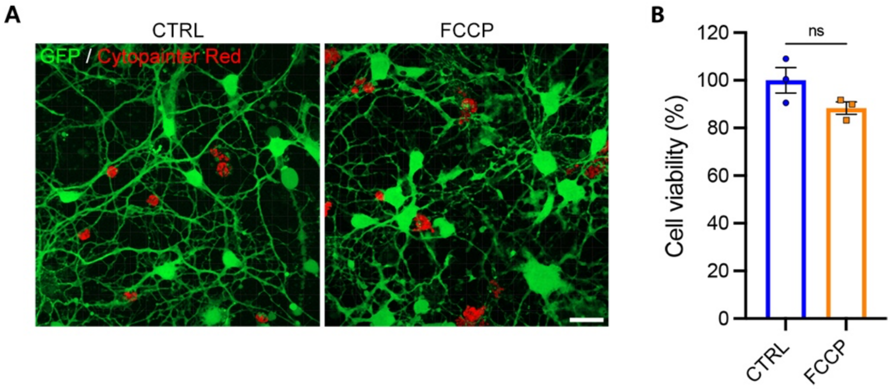

Fig. S3: Cell viability is not significantly impaired by treatment with mitochondria blocker FCCP for 40 minutes.
(**A**) Representative images of VCN neurons expressing cGFP (green) untreated (CTRL, left panel) and treated with 50 µM FCCP for 40 minutes (FCCP, right panel), and labeled with Cytopainter red dye (red) to detect dead cells, scale bar = 20 µm. (**B**) Quantification of cell viability in untreated (blue, CTRL) and FCCP-treated (orange, FCCP) neurons. Data are plotted as mean ± sem (n = 3 independent experiments), and statistical analysis was performed with unpaired Student's t-test. ns: not significant.

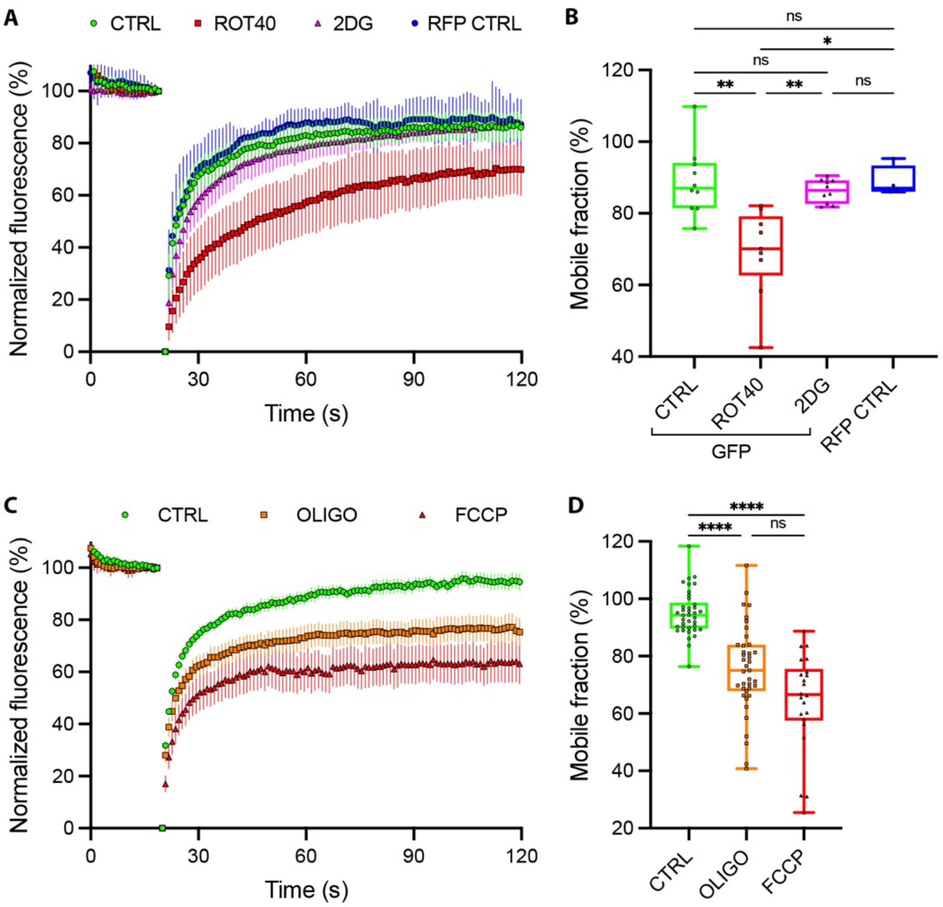

Fig. S4: Mitochondria activity, but not glycolysis, is responsible for the regulation of cGFP recovery in mCTs and presynaptic axons.

(**A**) Fluorescence intensity recovery profiles in cGFP untreated (green, CTRL), cGFP treated with 20 μM Rotenone for 40 mins (red, ROT40), cGFP treated with 2 mM 2DG (magenta, 2DG), and in FusionRed untreated (blue, RFP CTRL) terminals. Data are plotted as mean ± 95% CI. (**B**) Mobile fraction representing cytosolic fluidity estimated from the last 20 s of the fluorescence intensity profile in cGFP control (green, n = 10 cells from 3 independent experiments), cGFP rotenone-treated (red, n = 9 cells from 3 independent experiments), cGFP 2DG-treated (magenta, n = 10 cells from 3 independent experiments), and in RFP control (blue, n = 4 cells from 3 independent experiments) mCTs. Data are presented as box plot with median and minimum/maximum whiskers. Statistical analysis was performed with Kruskal-Wallis nonparametric test with Dunn's correction for multiple comparison. ns = not significant, *: p = 0.0128, **: p = 0.0024 and 0.0064. (**C**) Fluorescence intensity recovery profiles in cGFP untreated (green, CTRL), treated with 20 μM oligomycin (orange, OLIGO), and treated with 50 μM FCCP (red, FCCP) pre-synaptic axons. Data are plotted as mean ± 95% CI. (**D**) Mobile fraction representing cytosolic fluidity estimated from the last 20 s of the fluorescence intensity profile in cGFP control (green, n = 38 cells from 3 independent experiments), oligomycin-treated (orange, n = 39 cells from 3 independent experiments), and FCCP-treated (red, n = 22 cells from 3 independent experiments) pre-synaptic axons. Data are presented as box plot with median and minimum/maximum whiskers. Statistical analysis was performed with Kruskal-Wallis nonparametric test with Dunn's correction for multiple comparison. ns = not significant, ****: p < 0.0001.

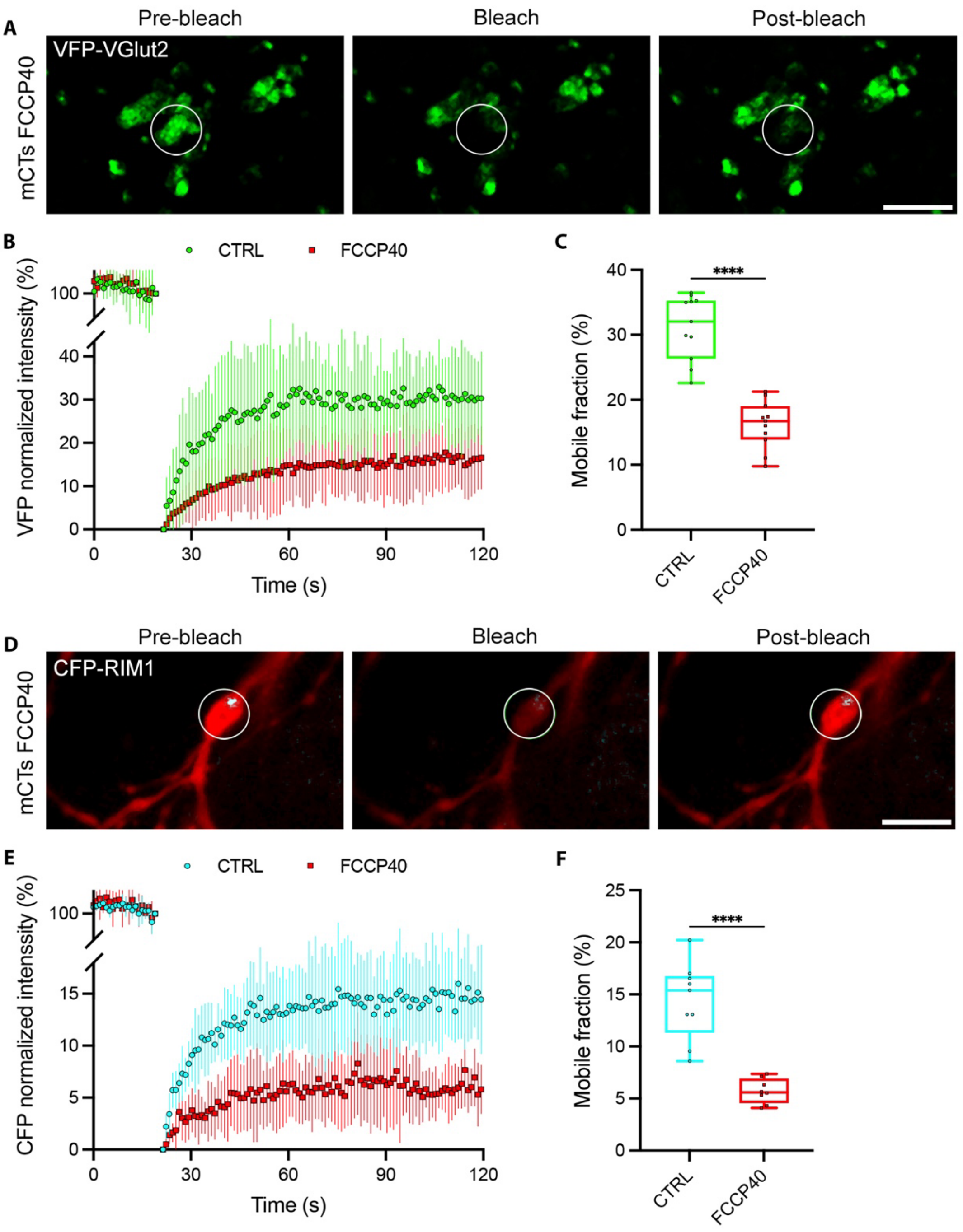

Fig. S5: Inhibition of mitochondria activity reduces synaptic vesicles and active zones fluid phase.
(**A**) Representative images of mouse calyceal terminals (mCTs) expressing Venus-VGLUT2 in culture for 18-21 days. FRAP was performed on ROI containing at least one calyceal swelling (white circle). Sequential images show ROI before bleach (left panel, at 10 s), during bleach (middle panel, at 22 s) and after recovery (right panel, at 120 s), scale bar = 5 µm. (**B**) VFP fluorescence intensity recovery profiles in control (green, n = 11 cells from 3 independent experiments) and in 50 µM FCCP treated mCTs for 40 mins (red, n = 11 cells from 3 independent experiments). Data are plotted as mean ± 95% CI. (**C**) Mobile Venus-VGLUT2 fraction representing SVs pool fluidity estimated from the last 20 s of the fluorescence intensity profile in control (green), and after 40 mins FCCP (red) treated mCTs. Data are presented as box plot with median and minimum/maximum whiskers. Statistical analysis was performed with Mann-Whitney nonparametric test. ****: $p < 0.0001$. (**D**) Representative images of mouse calyceal terminals (mCTs)

expressing cytosolic FusionRed and mTurqoise-RIM1 (CFP-RIM1) in culture for 18-21 days. FRAP was performed on ROI containing at least one calyceal swelling (white circle). Sequential images show ROI before bleach (left panel, at 10 s), during bleach (middle panel, at 22 s) and after recovery (right panel, at 120 s), scale bar = 5 μm. (**E**) CFP fluorescence intensity recovery profiles in control (green, n = 9 cells from 3 independent experiments) and in 50 μM FCCP treated mCTs for 40 mins (red, n = 8 cells from 3 independent experiments). Data are plotted as mean ± 95% CI. (**F**) Mobile mTurquoise-RIM1 fraction representing AZs fluidity estimated from the last 20 s of the fluorescence intensity profile in control (green), and after 40 mins FCCP (red) treated mCTs. Data are presented as box plot with median and minimum/maximum whiskers. Statistical analysis was performed with Mann-Whitney nonparametric test. ****: $p < 0.0001$.

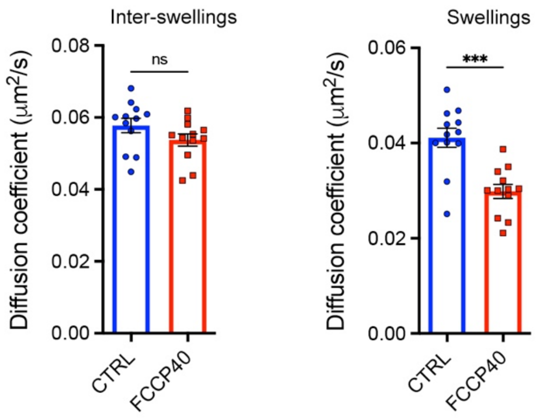

Fig. S6: FCCP treatment does not impair active transport of SVs between calyceal swellings but reduces their diffusive motion within swellings.

Diffusion coefficient (D) was estimated from MSD curves obtained after VGLUT2-SVs tracking as described in (*43*), in inter-swellings regions (left panel) and within swellings (right panel) in control condition (blue) or treated with FCCP for 40 mins (red, n = 12 cells from 3 independent experiments). Data are plotted as mean ± sem, and statistical analysis was performed with unpaired Student's t-test. ns: not significant, ***: p = 0.0002.

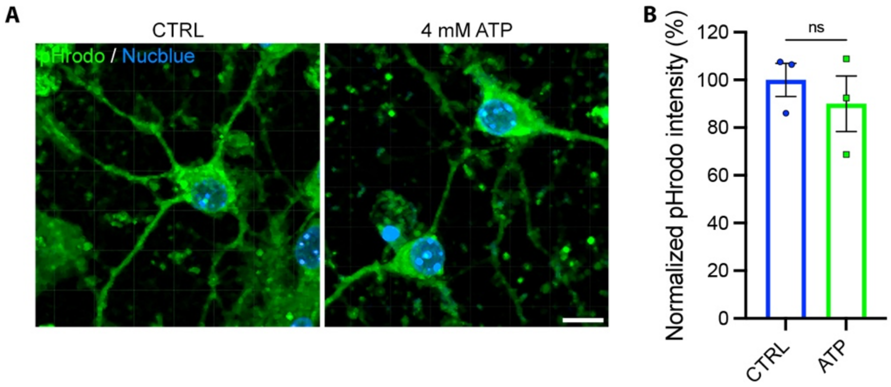

Fig. S7: Chitosan-mediated delivery of 4 mM ATP does not significantly affect cytosolic pH in VCN neurons. (**A**) Representative images of VCN neurons labeled with pHrodo (green) and Nucblue (blue) in control condition (CTRL, left panel) and after delivery of ATP (4 mM ATP, right panel), scale bar = 10 µm. (**B**) Quantification of pHrodo intensity in cell body of control VNC neurons (blue) and ATP loaded neurons (green). Data are plotted as mean ± sem (n = 3 independent experiments), and statistical analysis was performed with unpaired Student's t-test. ns: not significant.

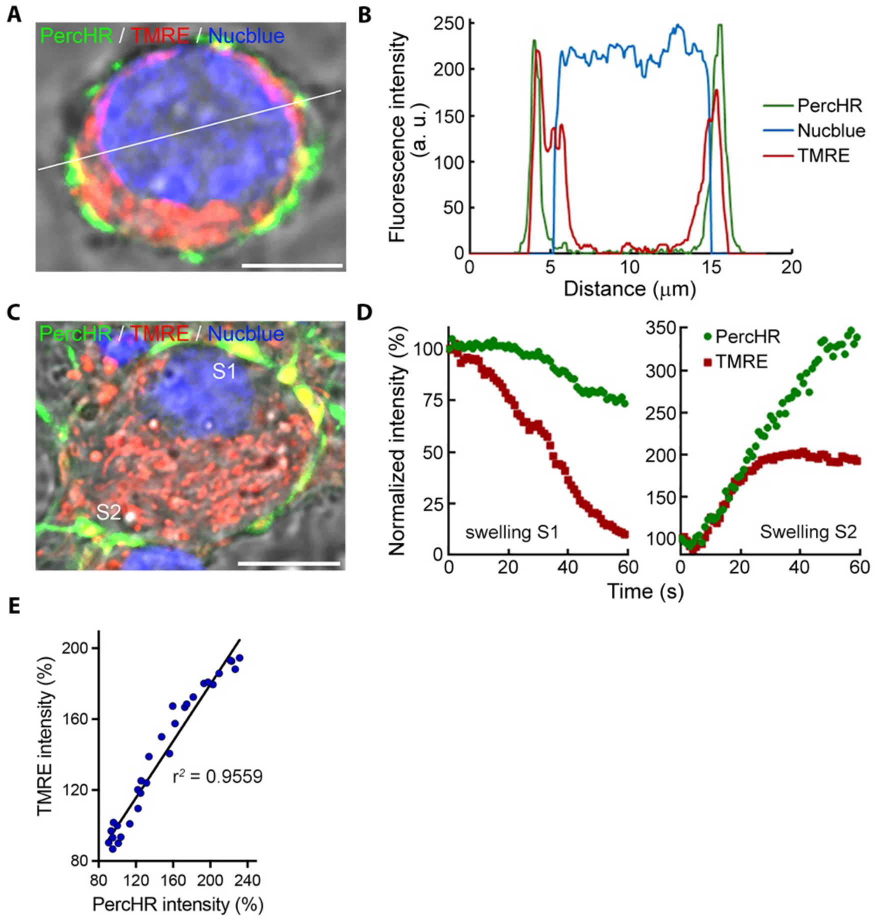

Fig. S8: Expression and localization of fluorescent ATP sensor PercevalHR in calyceal terminals. (**A**) Live confocal image of calyceal terminals expressing PercHR (green) and labeled with TMRE (red) and Nucblue (blue), scale bar = 10 μm. (**B**) Fluorescence intensity line profile from terminal shown in A. (**C**) Live confocal image of calyceal terminals expressing PercHR (green) and labeled with TMRE (red) and Nucblue (blue). Fluorescence intensity of TMRE and PercHR was monitored for 1 min in 2 individual swellings S1 and S2, scale bar = 10 μm. (**D**) Variation in the fluorescence of TMRE and PercHR in swelling S1 (left panel) and S2 (right panel). E) Correlation between TMRE and PercHR fluorescence intensity.

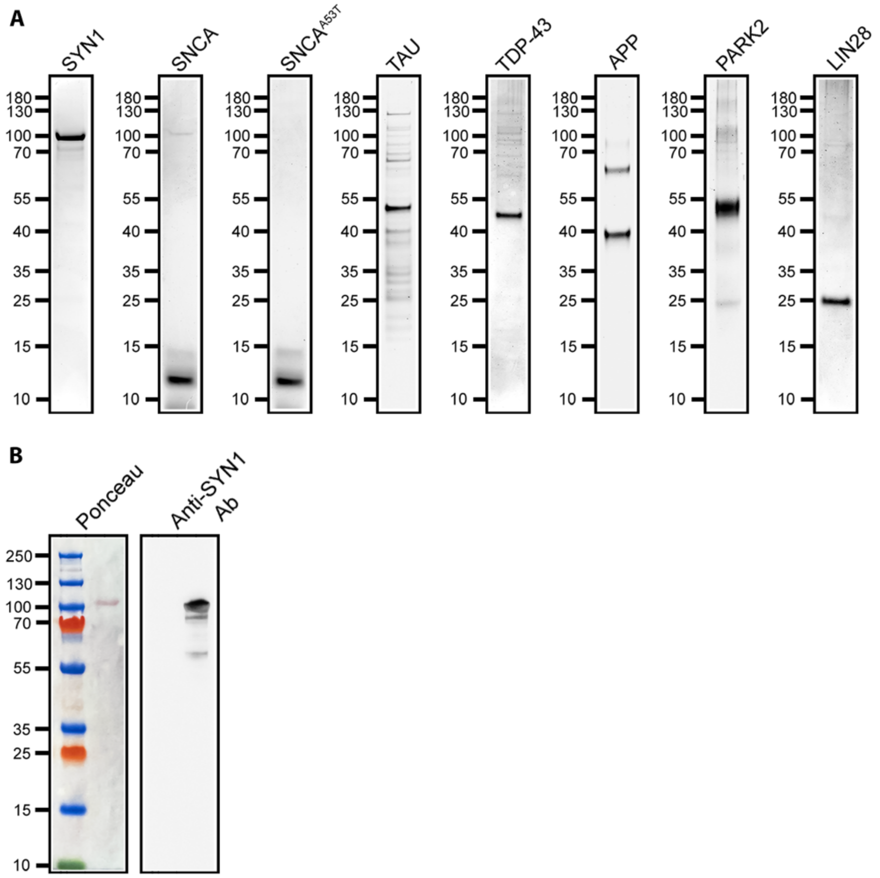

Fig. S9: SDS gel electrophoresis of purified proteins and western blot analysis of synapsin-1
(**A**) SDS-PAGE of purified proteins used in *in vitro* condensation assays. Proteins loaded into NuPage 4-12% Bis-Tris gel were visualized with colloidal blue staining. Molecular weight markers are shown in kDa for each protein. (**B**) Western blot of Synapsin-1 purified protein. Membrane transfer was detected by Ponceau staining (left panel) and the presence of Synapsin-1 was confirmed with anti-Synapsin-1 antibody (right panel). Molecular weight markers are shown in kDa.

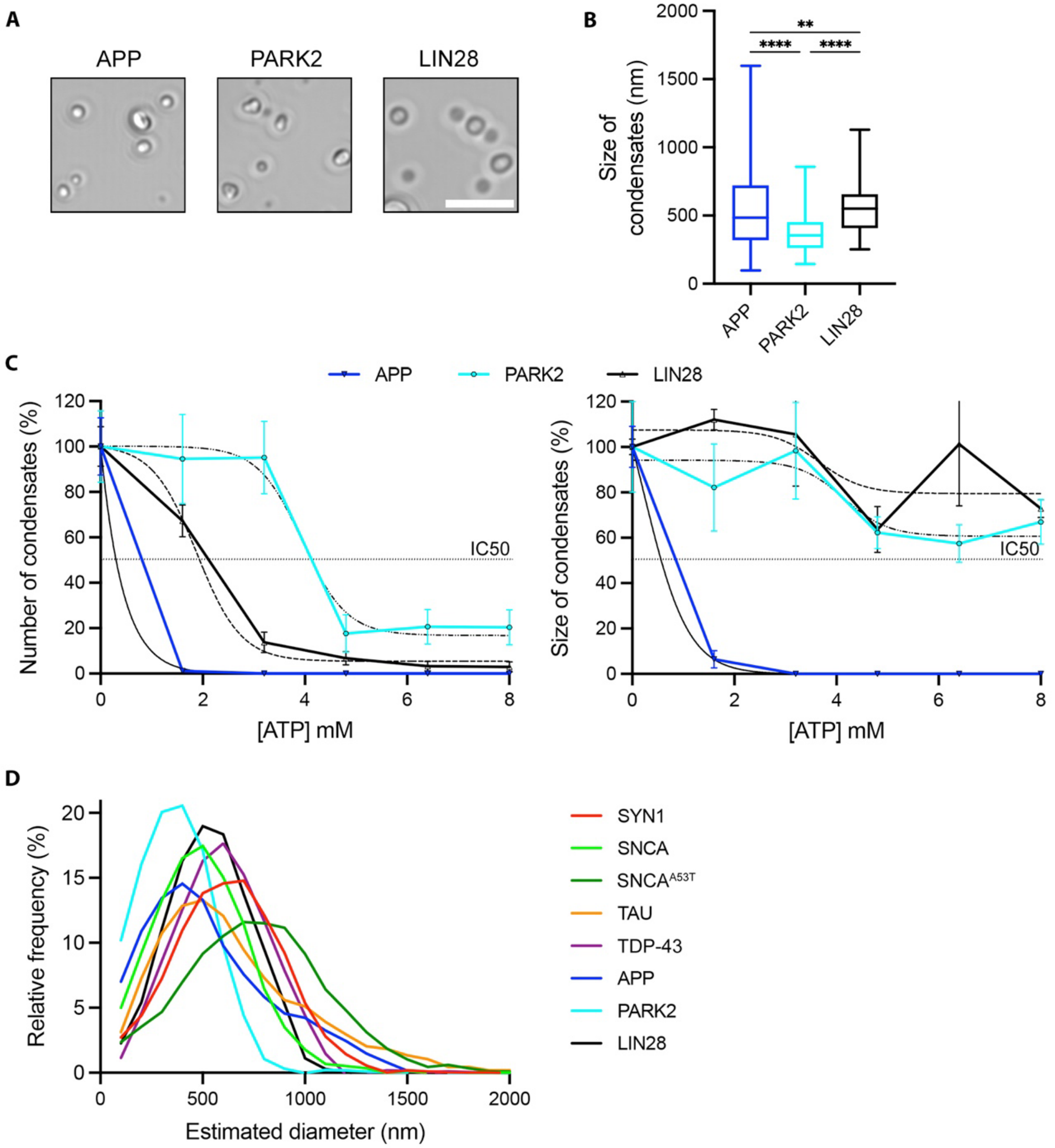

Fig. S10: ATP regulates neuronal proteins liquid phase separation *in vitro*.
(**A**) Representative images of liquid phase separated condensates formed after 1h at 37°C from 10 μM protein solutions, scale bar = 1 μm. (**B**) Estimated size of protein condensates obtained from binary images (n = 278, 284 and 275 aggregates for APP, Parkin (PARK2) and LIN28, respectively. Data are presented as box plot with median and minimum/maximum whiskers. Statistical analysis was performed with Kruskal-Wallis nonparametric test with Dunn's correction for multiple comparison. **: $p = 0.0086$, ****: $p < 0.0001$. (**C**) Proportion of remaining condensates (left panel) and size of remaining condensates (right panel) after addition of increasing concentration of ATP. Data are plotted as mean ± sem (pooled from 4 independent experiments). IC50 = half-maximal inhibition concentration. (**D**) Frequency distribution of condensate sizes for all proteins tested.

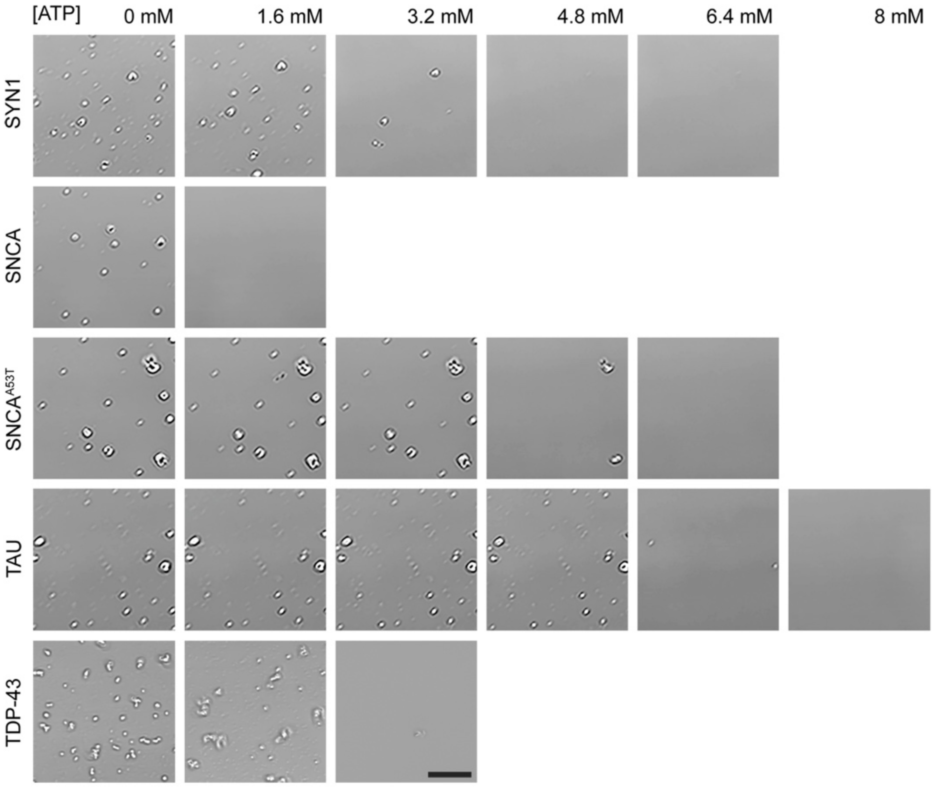

Fig. S11: ATP promotes solubilization of preformed condensates *in vitro*.
Live confocal imaging showing the solubilization of preformed protein condensates by ATP. 10 μM protein solutions were incubated for 1h at 37°C and further submitted to increasing concentration of ATP until complete de-condensation, scale bar = 4 μm.

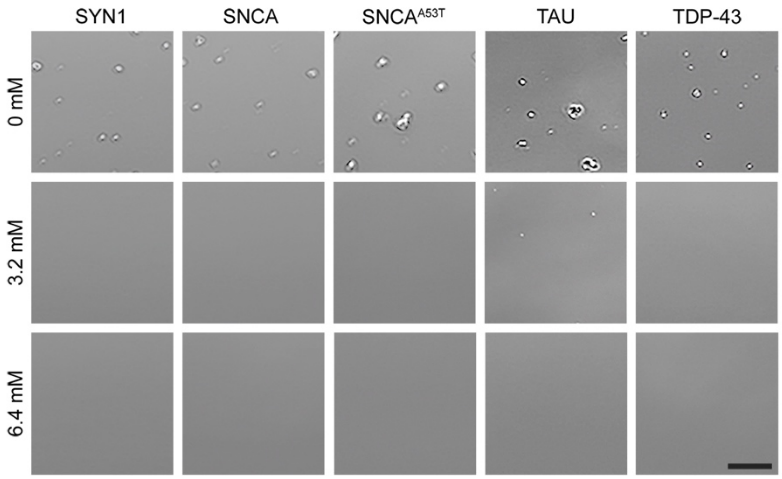

Fig. S12: ATP prevents formation of liquid phase separated condensates *in vitro*.
10 μM protein solutions were incubated in the absence or in the presence of ATP for 1h at 37°C. Protein condensate formation in control condition (no ATP, upper panels) and in the presence of 3.2 mM (middle panels) or 6.4 mM (lower panels) of ATP, scale bar = 5 μm.

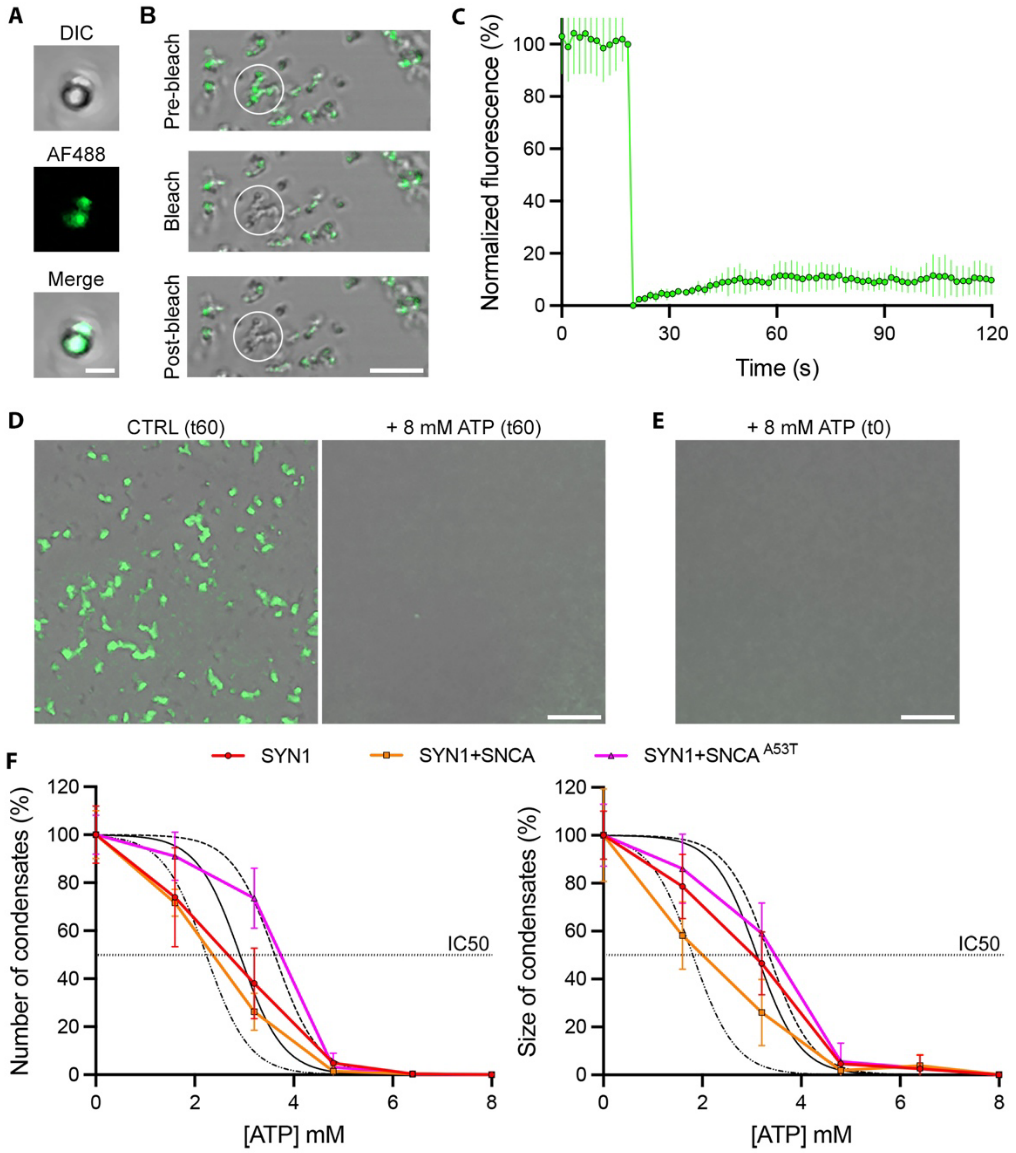

Fig. S13: *In vitro* FRAP analysis of SNCA^{A53T} droplets and ATP sensitivity of SYN1 mixed droplets.
(**A**) Representative image of fluorescently labelled SNCA^{A53T} phase separated droplet, scale bar = 1 µm. (**B**) Sequential images showing AF488-SNCA^{A53T} droplets before bleach (upper panel), during bleach (middle panel) and after recovery (right panel), scale bar = 5 µm. (**C**) Fluorescence recovery profile of AF488-SNCA^{A53T} droplets. Data are plotted as mean ± 95% CI (n = 10 samples from 3 independent experiments). (**D**) Representative images of AF488-SNCA^{A53T} droplets after 1 hour incubation before (left panel) and after (right panel) the addition of 8 mM ATP. (**E**) Representative image showing the absence of AF488-SNCA^{A53T} droplet formation in the presence of 8 mM ATP from the start of the 1-hour incubation period. Scale bars in **D** and **E** = 5 µm. (**F**) Proportion of remaining SYN1 (red), SYN1/SNCA (orange) and SYN1/SNCA^{A53T} (magenta) condensates (left panel) and their respective sizes (right panel) after addition of increasing concentration of ATP. Data are plotted as mean ± sem (pooled from 4 (SYN1) and 3 (SYN1/SNCA and SYN1/SNCA^{A53T}) independent experiments). IC50 = half-maximal inhibition concentration.

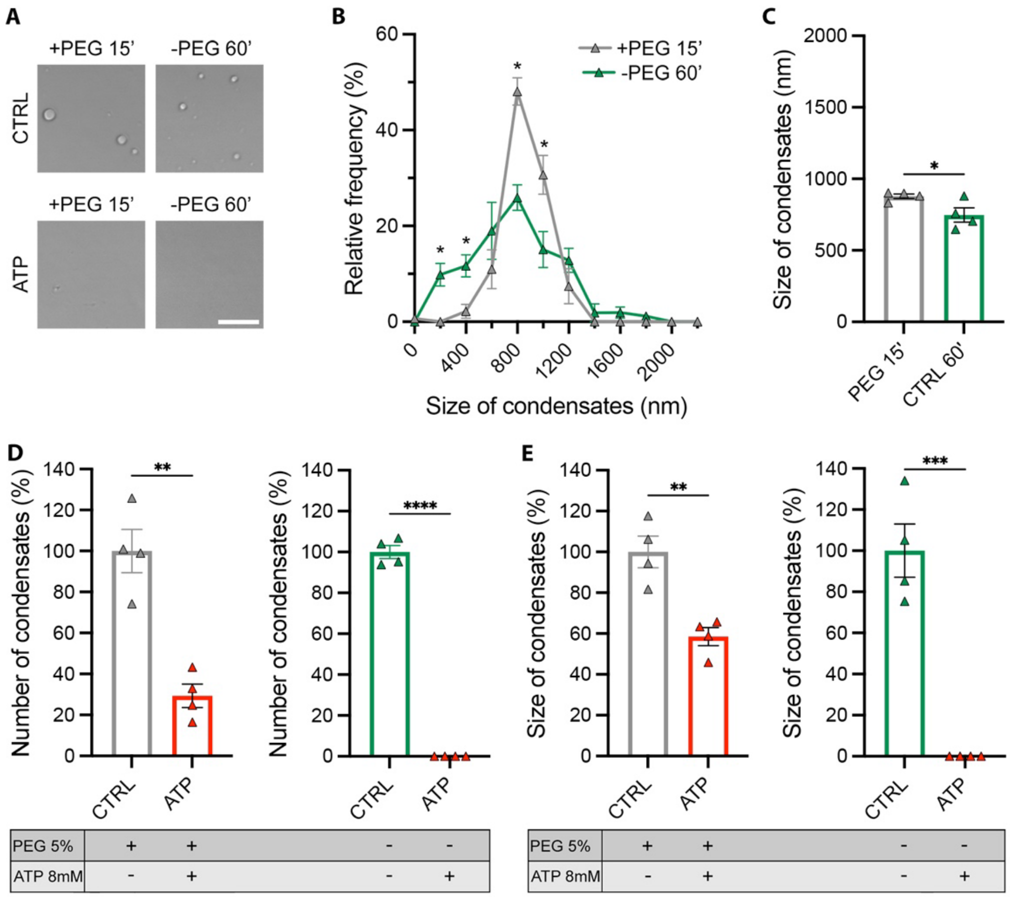

Fig. S14: ATP-dependent decondensation of SNCA^{A53T} droplets preformed in the presence of PEG.
(**A**) Representative images of liquid phase separated SYN1 condensates formed after 15' in the presence of 5% PEG and after 60' without PEG at 37°C from 10 µM protein solutions in control condition (upper panels) or after addition of 8 mM ATP (lower panels), scale bar = 5 µm. (**B**) Distribution of SNCA^{A53T} droplet sizes with (grey) and without (dark red) PEG. Data are plotted as mean ± sem (pooled from 4 independent experiments), and statistical analysis was performed with multiple unpaired Student's t-test. *: p = 0.0044, 0.0097, 0.0003, 0.0205. (**C**) Average size of SNCA^{A53T} droplets with (grey) and without (dark green) PEG. Data are plotted as mean ± sem (pooled from 4 independent experiments), and statistical analysis was performed with unpaired Student's t-test. *: p = 0.0471. (**D**) Normalized number of SNCA^{A53T} droplets with (left panel) and without (right panel) PEG in control conditions (grey and dark green) or after addition of ATP (red). Data are plotted as mean ± sem (pooled from 4 independent experiments), and statistical analysis was performed with unpaired Student's t-test. **: p = 0.0011, ****: p < 0.0001. (**E**) Normalized size of SNCA^{A53T} droplets with (left panel) and without (right panel) PEG in control conditions (grey and dark green) or after addition of ATP (red). Data are plotted as mean ± sem (pooled from 4 independent experiments), and statistical analysis was performed with unpaired Student's t-test. *: p = 0.0035, ***: p = 0.0002.

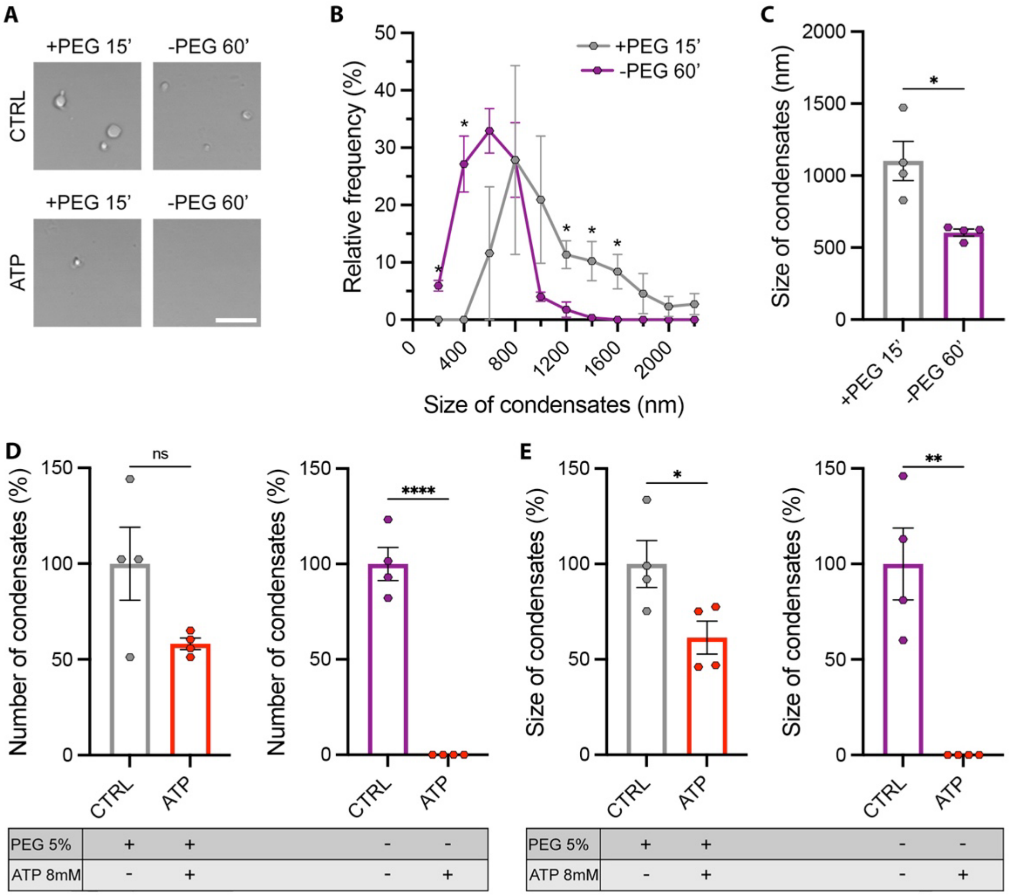

Fig. S15: ATP-dependent decondensation of TDP-43 droplets preformed in the presence of PEG.
(**A**) Representative images of liquid phase separated SYN1 condensates formed after 15' in the presence of 5% PEG and after 60' without PEG at 37°C from 10 μM protein solutions in control condition (upper panels) or after addition of 8 mM ATP (lower panels), scale bar = 5 μm. (**B**) Distribution of TDP-43 droplet sizes with (grey) and without (purple) PEG. Data are plotted as mean ± sem (pooled from 4 independent experiments), and statistical analysis was performed with multiple unpaired Student's t-test. *: p = 0.0001, 0.0004, 0.0146, 0.0395, 0.0430. (**C**) Average size of TDP-43 droplets with (grey) and without (purple) PEG. Data are plotted as mean ± sem (pooled from 4 independent experiments), and statistical analysis was performed with unpaired Student's t-test, *: p = 0.0111. (**D**) Normalized number of TDP-43 droplets with (left panel) and without (right panel) PEG in control conditions (grey and purple) or after addition of ATP (red). Data are plotted as mean ± sem (pooled from 4 independent experiments), and statistical analysis was performed with unpaired Student's t-test. ns: p = 0.0728, ****: p < 0.0001. (**E**) Normalized size of TDP-43 droplets with (left panel) and without (right panel) PEG in control conditions (grey and purple) or after addition of ATP (red). Data are plotted as mean ± sem (pooled from 4 independent experiments), and statistical analysis was performed with unpaired Student's t-test, *: p = 0.042, **: p < 0.002.

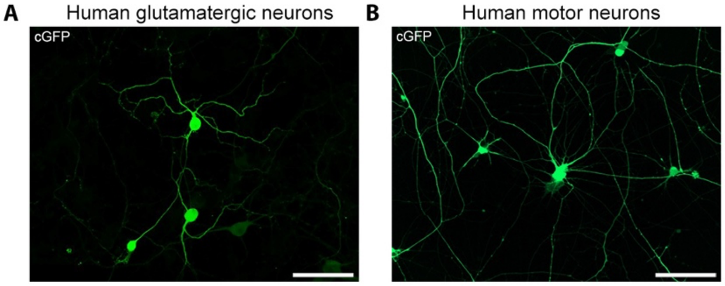

Fig. S16: Live imaging of human iPSC-derived neurons in culture.
(**A**) Healthy control human glutamatergic neurons (hGNs) transfected by electroporation and expressing cGFP after 15 days in culture. (**B**) Healthy control human motor neurons (hMNs) transfected with AAV and expressing cGFP after 18 days in culture. Scale bars in **A** and **B** = 50 μm.

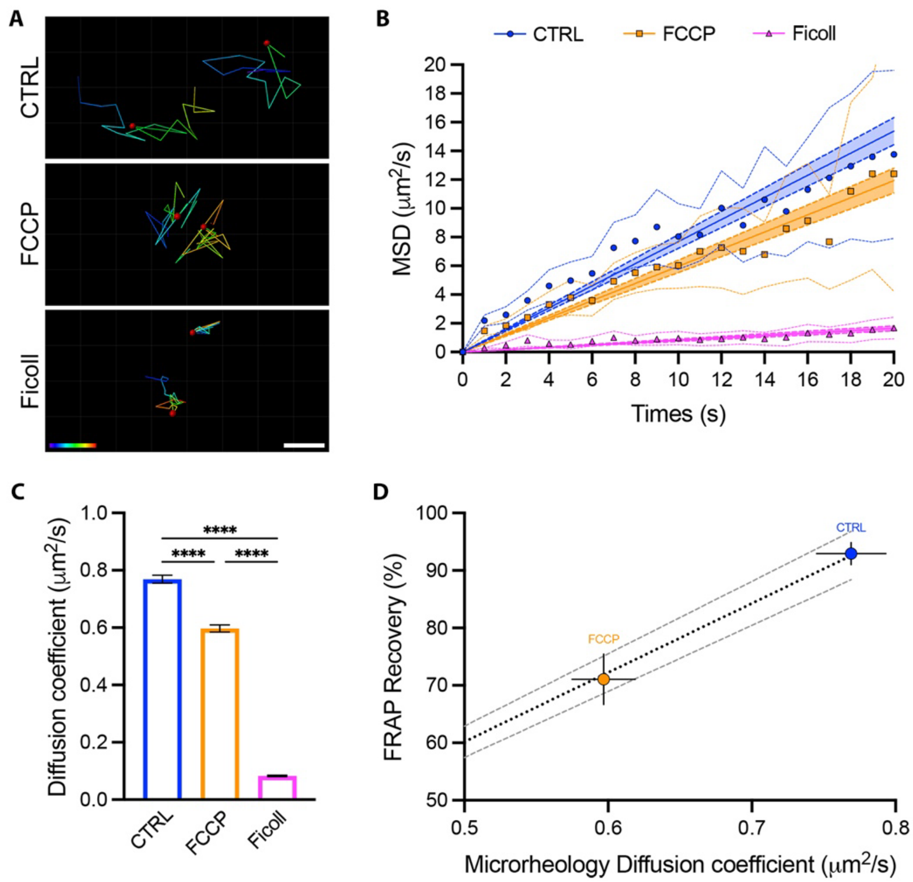

Fig. S17: *In cellulo* FRAP analysis correlates with viscosity measured *in vitro* by microrheology on purified cytosol.

(**A**) Representative images of rhodamine-labelled nanobead tracking in purified cytosolic fraction from untreated (CTRL) and FCCP-treated (FCCP) human motor neurons, or in 15% Ficoll 400 solution (Ficoll). Tracks are color-coded according to time, scale bar = 2 μm, color bar = 0 to 30 seconds. (**B**) Mean square displacement plots calculated from identified tracks in untreated cytosol (blue dots), FCCP-treated cytosol (orange squares) and 15% Ficoll (magenta triangles) and their corresponding linear regressions. Dashed lines represent 95% CI (n = 58, 48 and 39 tracks for CTRL, FCCP and Ficoll samples respectively from 3 independent experiments). (**C**) Diffusion coefficient calculated from the linear regression plots for untreated cytosol (blue, CTRL), FCCP-treated cytosol (orange, FCCP) and 15% Ficoll (magenta, Ficoll). Data are plotted as mean ± sem, and statistical analysis was performed with one-way ANOVA with Tukey's correction for multiple comparison. ****: $p < 0.0001$. (**D**) Correlation between diffusion coefficients from microrheology analysis and cGFP fluorescence recoveries from FRAP analysis in untreated control (blue) and FCCP-treated (orange) samples. Data are plotted as mean ± sem with simple linear regression (dotted line, $r^2 = 0.99$) and 95% CI (dashed lines).

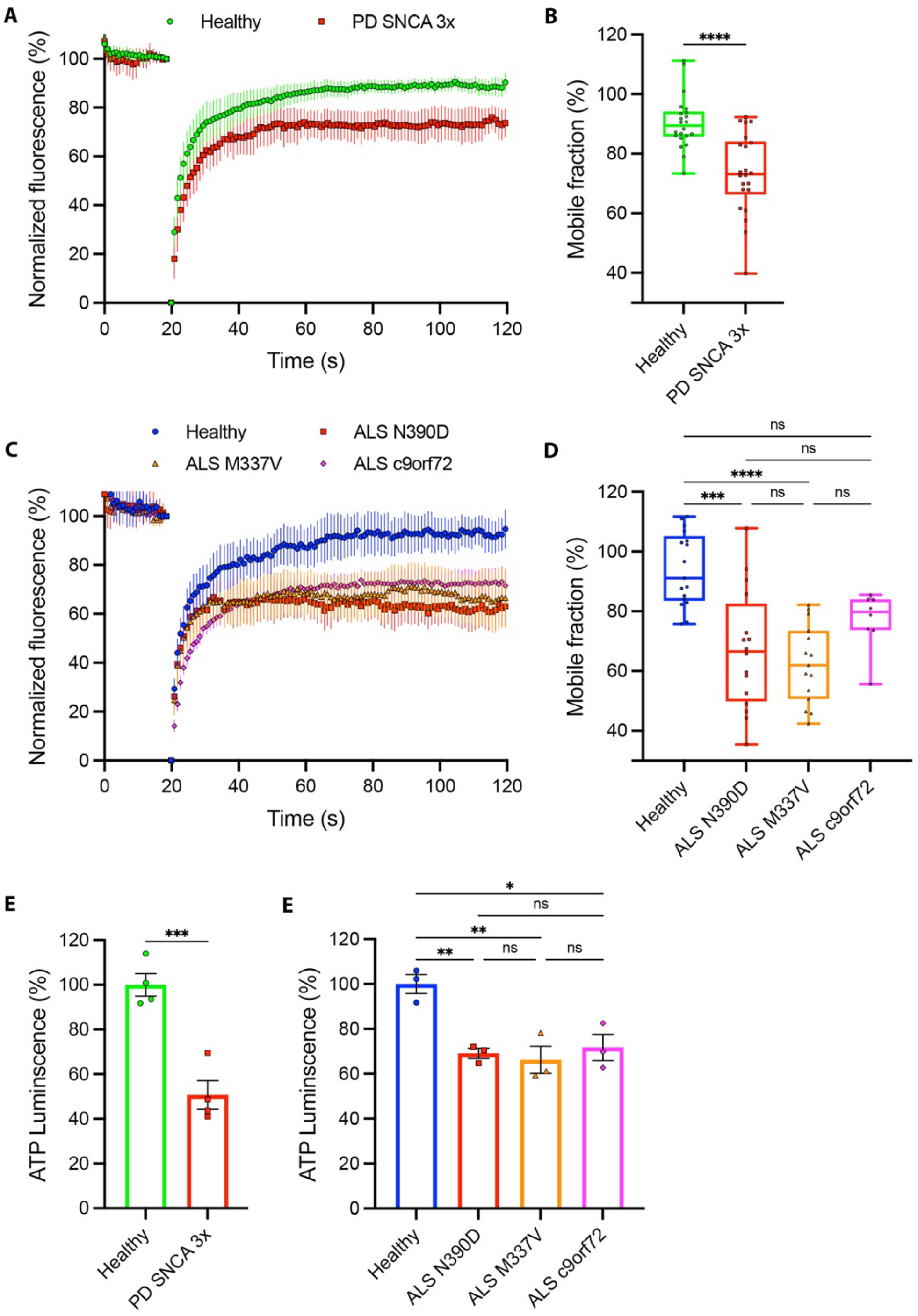

Fig. S18: Reduced axonal cytosol viscoelasticity and ATP levels in PD and ALS neurons.
(**A**) Fluorescence intensity recovery profiles in healthy control (green) and in PD SNCA 3x (red) patient hGNs. Data are plotted as mean ± 95% CI. (**B**) Mobile cGFP fraction representing cytosolic fluidity estimated from the last 20 s of the fluorescence intensity profile in healthy control (green, n = 22 cells from 4 independent experiments) and PD SNCA 3x (red, n = 22 cells from 4 independent experiments) patient hGNs. Data are presented as box plot with median and minimum/maximum whiskers. Statistical analysis was performed with Mann-Whitney nonparametric test. ****: $p < 0.0001$. (**C**) Fluorescence intensity recovery profiles in healthy control (blue), ALS TDP-43^{N390D} (red), TDP-43^{M337V} (orange), and c9orf72 HRE (magenta) patient hMNs. Data are plotted as mean ± 95% CI. (**D**) Mobile cGFP fraction representing cytosolic fluidity estimated from the last 20 s of the fluorescence intensity profile in healthy

control (blue, n = 16 cells from 3 independent experiments), ALS TDP-43^{N390D} (red, n = 16 cells from 3 independent experiments), TDP-43^{M337V} (orange, n = 15 cells from 3 independent experiments) and c9orf72 HRE (magenta, n = 9 cells from 3 independent experiments) patient hMNs. Data are presented as box plot with median and minimum/maximum whiskers. Statistical analysis was performed with Kruskal-Wallis nonparametric test with Dunn's correction for multiple comparison. ns: not significant, ***: $p = 0.0003$, ****: $p < 0.0001$. (**E**) *In vitro* bioluminescence measurement of intracellular ATP in healthy control (green) and PD SNCA 3x patient (red) hGNs. Intracellular level of ATP was measured after cell lysis in CellTiterGlo 2.0 reagent (from 4 independent experiments). Data are plotted as mean ± sem, and statistical analysis was performed with unpaired Student's t-test. ***: $p = 0.0010$. F) *In vitro* bioluminescence measurement of intracellular ATP in healthy control (blue) ALS TDP-43^{N390D} (red), TDP-43^{M337V} (orange) and c9orf72 HRE (magenta) patient hMNs. Intracellular level of ATP was measured after cell lysis in CellTiterGlo 2.0 reagent (from 3 independent experiments). Data are plotted as mean ± sem, and statistical analysis was performed with one-way ANOVA with Tukey's correction for multiple comparison. ns: not significant, *: $p = 0.0139$, **: $p = 0.0084$ and 0.0050.

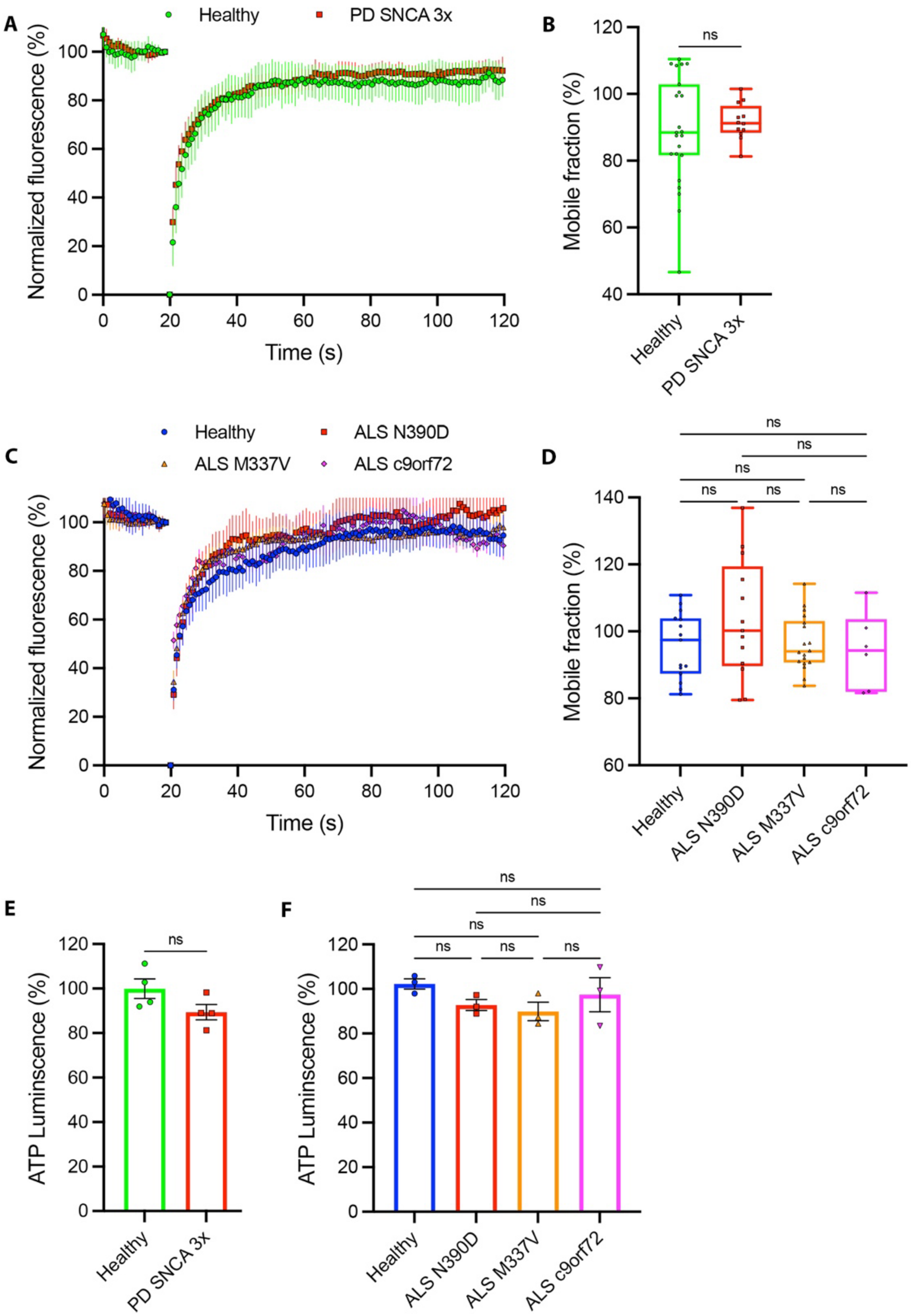

Fig. S19: NMN chronic treatment rescues axoplasmic viscosity and ATP levels in PD and ALS neurons.
(**A**) Fluorescence intensity recovery profiles in healthy control (green) and in PD SNCA 3x patient (red) hGNs after chronic NMN treatment. Data are plotted as mean ± 95% CI. (**B**) Mobile cGFP fraction representing cytosolic fluidity estimated from the last 20 s of the fluorescence intensity profile in healthy control (green, n = 23 cells from 4 independent experiments) and PD SNCA 3x (red, n = 12 cells from 4 independent experiments) patient hGNs. Data are presented as box plot with median and minimum/maximum whiskers. Statistical analysis was performed with Mann-Whitney nonparametric test. (**C**) Fluorescence intensity recovery profiles in healthy control (blue), ALS TDP-43^{N390D} (red), TDP-43^{M337V} (orange), and c9orf72 HRE (magenta) patient hMNs. Data are plotted as mean ± 95% CI. (**D**) Mobile cGFP fraction representing cytosolic fluidity estimated from the last 20 s of the fluorescence intensity

profile in healthy control (blue, n = 15 cells from 3 independent experiments), ALS TDP-43^{N390D} (red, n = 13 cells from 3 independent experiments), TDP-43^{M337V} (orange, n = 18 cells from 3 independent experiments) and c9orf72 HRE (magenta, n = 6 cells from 3 independent experiments) patient hMNs. Data are presented as box plot with median and minimum/maximum whiskers. Statistical analysis was performed with Kruskal-Wallis nonparametric test with Dunn's correction for multiple comparison. (**E**) *In vitro* bioluminescence measurement of intracellular ATP in healthy control (green) and PD SNCA 3x (red) patient hGNs. Intracellular level of ATP was measured after cell lysis in CellTiterGlo 2.0 reagent (from 4 independent experiments). Data are plotted as mean ± sem, and statistical analysis was performed with unpaired Student's t-test. (**F**) *In vitro* bioluminescence measurement of intracellular ATP in healthy control (blue), ALS TDP-43^{N390D} (red), TDP-43^{M337V} (orange) and c9orf72 HRE (magenta) patient hMNs. Intracellular level of ATP was measured after cell lysis in CellTiterGlo 2.0 reagent (from 3 independent experiments). Data are plotted as mean ± sem, and statistical analysis was performed with one-way ANOVA with Tukey's correction for multiple comparison. ns: not significant in panels **B**, **D**, **E** and **F**.

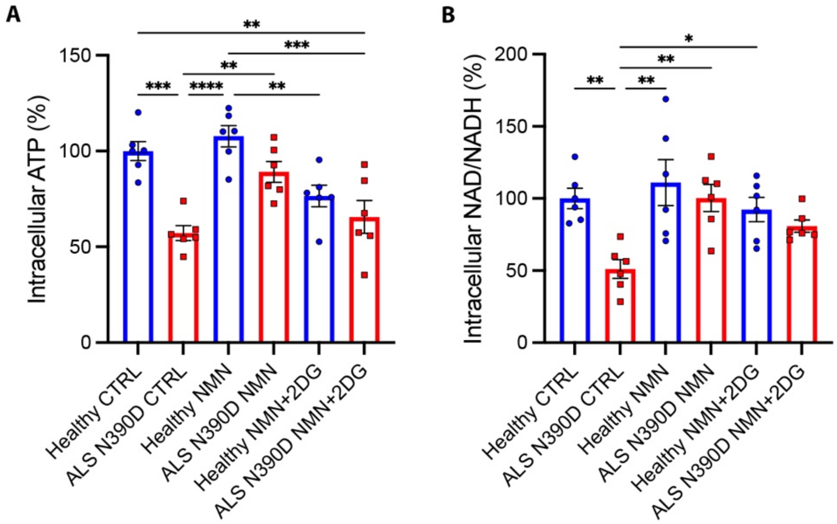

Fig S20: Glycolysis inhibition reduces NMN-induced ATP synthesis in ALS TDP-43^{N390D} motor neurons.
(**A**) *In vitro* bioluminescence measurement of intracellular ATP in healthy control (blue) and ALS TDP-43^{N390D} (red) patient hMNs. Cells were untreated (CTRL), treated with NMN (NMN) or with NMN and glycolysis inhibitor 2DG (NMN+2DG). Intracellular level of ATP was measured after cell lysis in CellTiterGlo 2.0 reagent (from 6 independent experiments). Data are plotted as mean ± sem, and statistical analysis was performed with one-way ANOVA with Tukey's correction for multiple comparison. **: p = 0.0030, 0.0066 and 0.0082, ***: p = 0.0002, ****: p < 0.0001.
(**B**) *In vitro* bioluminescence measurement of intracellular NAD/NADH in healthy control (blue) and ALS TDP-43^{N390D} (red) patient hMNs. Cells were not treated (CTRL), treated with NMN (NMN) or with NMN and glycolysis inhibitor 2DG (NMN+2DG). Intracellular level of NAD/NADH was measured after cell lysis of cells in NAD/NADH-Glo reagent (from 6 independent experiments). Data are plotted as mean ± sem, and statistical analysis was performed with one-way ANOVA with Tukey's correction for multiple comparison. *: p = 0.0406, **: p = 0.0097, 0.001 and 0.0091.

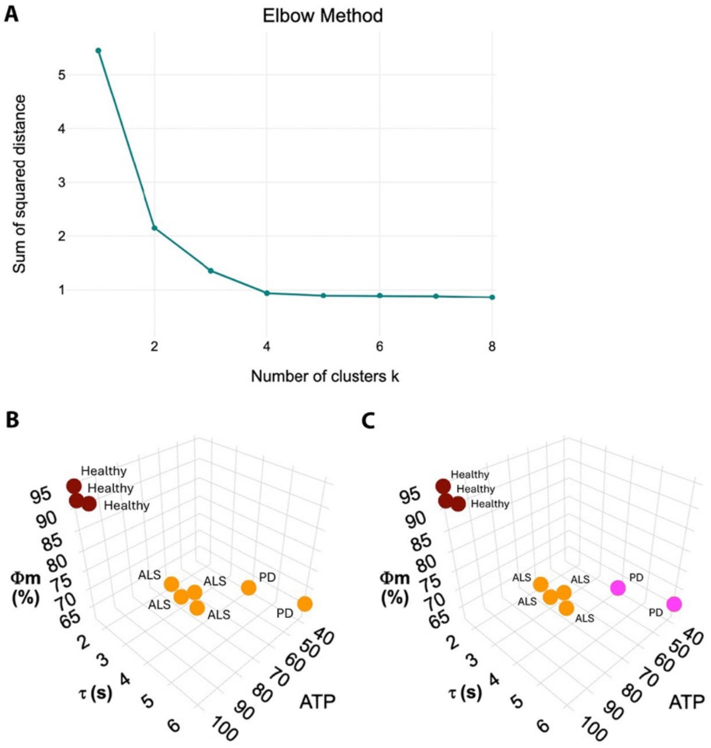

	ATP (%)	τ (s)	Φm (%)
Healthy	100	1.8	93.33
Diseases (ALS +PD)	64.40	4.16	67.16

	ATP (%)	τ (s)	Φm (%)
Healthy	100	1.8	93.33
ALS	69.75	3.23	66
PD	49.5	5.6	69.5

Fig S21: Viscosity and ATP measurements cluster healthy versus disease neurons.
(**A**) Determination of the optimal number of clusters by the Elbow method. (**B**) k-means cluster analysis with 2 clusters, healthy (red) and diseases (orange, PD+ALS). (**C**) k-means cluster analysis with 3 clusters, healthy (red), PD (magenta) and ALS (orange). Cluster centroids are indicated in tables for panels **B** and **C** (variables: ATP level, mobile fraction Φm, and half recovery time τ).

Line	Type	Mutation	Φm (%)	τ (s)	ATP (%)
**HPS0077**	Healthy	-	95 (89)	1.6 (3)	100 (96)
**HSP0097**	PD	PARK2	66 (84)	6.1 (3.7)	39 (83)
**STBCi024-A**	PD	SNCA 3x	73 (88)	5.1 (2)	60 (92)
**CS9LL4iCTR-nxx**	Healthy	-	93 (94)	2.2 (1.9)	100 (113)
**CS5ZLDiALS-nxx**	ALS	TDP-43^{N390D}	66 (90)	3.9 (2.3)	72 (89)
**CS2PFYiCTR-nxx**	Healthy	-	92 (97)	1.6 (2.2)	100 (102)
**CS1UWUiALS-nxx**	ALS	TDP-43^{N390D}	66 (96)	2.5 (1.9)	69 (93)
**CS3MG8iALS-nxx**	ALS	TDP-43^{M337V}	62 (101)	2.7 (2.3)	66 (90)
**CS29iALS-C9nxx**	ALS	C9orf72 HRE	70 (93)	3.8 (2.4)	72 (97)

Table S1: Summary of axoplasmic viscosity and ATP levels in PD and ALS iPSC-derived neurons.
Cytosol viscosity in axons was quantified by the cGFP mobile fraction (Φm) and the half recovery time (τ) determined by FRAP analysis. Intracellular ATP levels were measured by *in vitro* luminescence assay. The values obtained after NMN treatment are shown in parenthesis. Altogether, disease lines show a 33% reduction in Φm and a 2.2x increase in τ, as well as a 37% decrease in ATP levels compared to healthy lines.
